# Polymorph Screening of Core-Chlorinated Naphthalene Diimides with Different Fluoroalkyl Side-Chain Lengths

**DOI:** 10.3390/molecules29184376

**Published:** 2024-09-14

**Authors:** Inês de Oliveira Martins, Marianna Marchini, Lucia Maini, Enrico Modena

**Affiliations:** 1 Department of Chemistry “Giacomo Ciamician”, University of Bologna, Via Selmi 2, 40126 Bologna, Italy; ines.deoliveira2@unibo.it; 2 PolyCrystalLine SPA, Via Della Cooperazione 29, 40059 Medicina, Italy; enrico.modena@polycrystalline.it

**Keywords:** organic semiconductor, polymorph, NDI, OFET, n-type semiconductor

## Abstract

In this work, naphthalenediimide (NDI) derivatives are widely studied for their semiconducting properties and the influence of the side-chain length on the crystal packing is reported, along with the thermal properties of three core-chlorinated NDIs with different fluoroalkyl side-chain lengths (CF_3_-NDI, C_3_F_7_-NDI and C_4_F_9_-NDI). The introduction of fluorinated substituents at the imide nitrogen and addition of strong electron-withdrawing groups at the NDI core are used to improve the NDI derivatives air stability. The new compound, CF_3_-NDI, was deeply analyzed and compared to the well-known C_3_F_7_-NDI and C_4_F_9_-NDI, leading to the discovery and solution of two different crystal phases, form α and solvate form, and a solid solution of CF_3_-NDI and CF_3_-NDI-OH, formed by the decomposition in DMSO.

## 1. Introduction

Organic field-effect transistors (OFETs) hold significant potential as essential components in the fabrication of low-cost, large-area, and flexible electronic devices [1,2,3,4]. The development of electronic circuits using organic semiconductors requires complementary logic elements, beyond individual transistors, such as voltage inverters, which are fundamental for circuit operation. These components demand n-type and p-type semiconductors, which are crucial for the achievement of low power dissipation, stable operation in circuits, and high-speed performance [5,6,7]. Even though substantial advancements have been made in the development of p-type organic semiconductors, it remains challenging to obtain n-type semiconductors that exhibit high charge-carrier mobility and air stability [4,7,8,9,10,11,12]. Indeed, the primary hurdle in developing efficient n-type semiconductors lies in the identification of materials that simultaneously show high electron mobility and air stability: this challenge arises from the high sensitivity of electrons to ambient oxidants such as oxygen and moisture, which act as electron traps, impairing device performance [9,12,13,14,15,16].

To overcome these obstacles, two molecular design strategies have emerged: the first strategy involves the incorporation of strong electron-withdrawing groups, such as cyano (-CN), chloro (-Cl), and fluoro (-F) groups, at lateral core positions of the semiconductor molecules. This approach effectively reduces the lowest unoccupied molecular orbital (LUMO) energy level, thereby lowering the electron injection barrier and creating a barrier to the penetration of oxygen and moisture, enhancing the air stability of the devices. The second approach involves the introduction of fluoroalkyl chains as substituents at the imide nitrogen. These chains form a sterically packed barrier that further hinders the reaction with atmospheric oxygen and moisture, thus improving the long-term stability of the semiconductor [7,14,15,17].

Among n-type organic semiconductors, naphthalene tetracarboxylic diimides (NDIs) are considered highly promising due to their high electron affinity, extensive π-orbital overlap in the solid state, and tunable optoelectronic properties, and they can be easily obtain from commercially available precursors [9,14,18,19,20]. When the NDI derivative is functionalized with fluoroalkyl chains at the imide nitrogen positions and chlorine substituents on the core, it exhibits considerable potential as an n-type organic semiconductor. Indeed, the incorporation of fluoroalkyl chains significantly influences the molecular packing and, in turn, the charge carrier mobility of NDIs [4,9].

The molecular packing of organic small molecule materials is strongly related to charge-carrier transport properties, since close molecular packing with π-orbital overlaps between neighboring molecules improves the charge carrier transport and facilitates high mobility [21,22,23]. Even subtle alterations in the packing motif or the relative orientation of molecules can lead to significant variations in charge carrier mobility [7,12,17,24].

Organic molecular materials are inclined to crystallize into different polymorphs with various packing arrangements as a consequence of the noncovalent interactions [25,26,27]. Therefore, the performance of organic devices is determined not only by the intrinsic molecular properties but also by the specific crystal packing. It has been observed that different polymorphs of the same material can display varied mobilities. For that reason, it is essential to screen all accessible polymorphs through recrystallization and deposition techniques while assessing their relative stability and conductivity. This comprehensive understanding allows the selection of the most favorable crystal phase for device optimization [26,28,29].

**Scheme 1:**
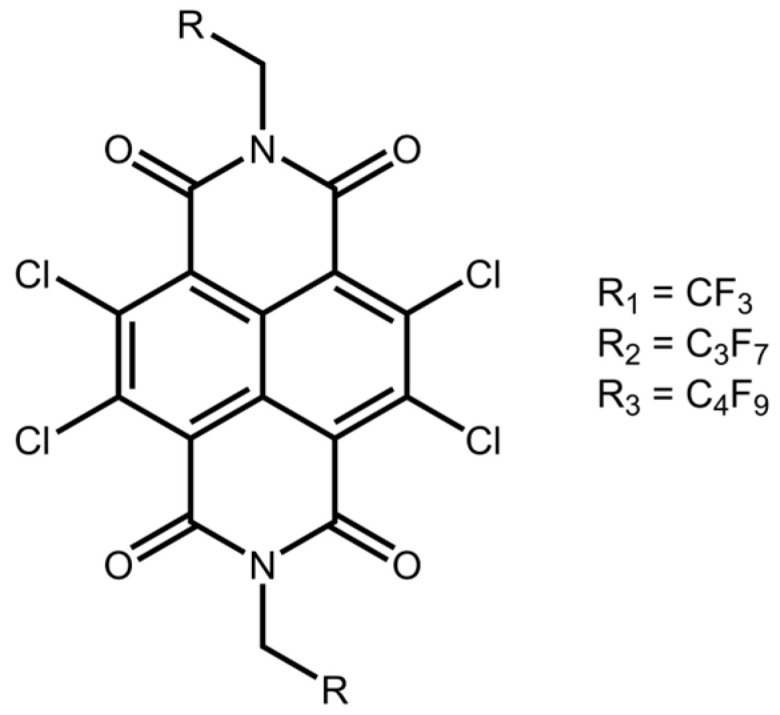
Molecular structure of studied NDI derivatives CF_3_-NDI (4,5,9,10-tetrachloro-2,7-bis (2,2,2-trifluoroethyl) benzo[lmn][3,8] phenanthroline-1,3,6,8(2H,7H)-tetraone), C_3_F_7_-NDI, and C_4_F_9_-NDI.

Given the critical role of crystal packing in determining the performance of organic molecular materials, the aim of our work is to investigate the polymorphic landscape of CF_3_-NDI, C_3_F_7_-NDI, and C_4_F_9_-NDI (see molecular structure in Scheme 1) by recrystallization in different solvents and by thermal characterization. C_3_F_7_-NDI and C_4_F_9_-NDI crystal structures are already known, along with their conductivity properties; however, there is no evidence that polymorph screening has been conducted. As for CF_3_-NDI, the crystal structure has never been reported so far, and a complete characterization study has been conducted for the first time. Indeed, we determined the crystal structures of three different crystal phases based on CF_3_-NDI and compared them with the structure of well-known compounds C_3_F_7_-NDI and C_4_F_9_-NDI, which have been deeply studied as an active layer of organic field effect transistors [4,12,14].

The polymorph screening on C_3_F_7_-NDI and C_4_F_9_-NDI revealed the presence of two new elusive crystal phases, and by thermal characterization we detected their enatiotropically related polymorphs, which are stable only at high temperature.

The presence of different polymorphs is important to determine the crystal phase, which can lead to more performative devices, or to avoid crystal phases with poor properties.

## 2. Results and Discussion

### 2.1. Polymorph Screening

The solubility assessment in several organic solvents, as reported in Table S1, shows a decrease in the solubility upon increasing the length of fluoroalkyl side chains. This particular feature is related to the low surface free energies of the fluoroalkyl chains and their poor polarizability (see Table S1). In the case of CF_3_-NDI, recrystallization experiments by solvent evaporation yielded always to the CF_3_-NDI Form α (starting material), except in DMSO and paraxylene (PXY). With solvent evaporation in PXY, we obtained a solvate called CF_3_-NDI·PXY, which converts into CF_3_-NDI Form α in a couple of hours at room conditions. When CF_3_-NDI is recrystallized in DMSO, both by solvent evaporation and by thermal gradient precipitation with a slow cooling profile, big orange block crystals were obtained and only after some effort were identified as a solid solution of CF_3_-NDI and a new molecule obtained by decomposition of CF_3_-NDI. The characterization of the new molecule was not straightforward, since it is always obtained as a solid solution with the starting compound; however, by single-crystal X-ray diffraction, ^13^C-NMR, ^1^H-NMR, and FTIR (Figures S1–S3), we were able to identify the new molecule as 4,5,9-trichloro-10-hydroxy-2,7-bis(2,2,2-trifluoroethyl)benzo[lmn][3,8] phenanthroline-1,3,6,8(2H,7H)-tetraone (CF_3_-NDI-OH). One of the chlorine atoms was substituted by the hydroxyl group, probably due to the presence of water in the DMSO. The substitution of the halogen atom was not expected since it happened during a simple recrystallization process, whereas it is commonly obtained in the presence of a reducing agent such as tetrabutylammonium fluoride (TBAF) [30].

It is worth noting that the crystals of the solid solution of CF_3_-NDI with CF_3_-NDI-OH (hereafter CF_3_-NDI·SS) are not isomorphic with CF_3_-NDI Form α, so we tried to seed a solution of CF_3_-NDI with a crystal of CF_3_-NDI∙SS to obtain a new polymorph, without any success.

Analogous polymorph screening was performed for C_3_F_7_-NDI and C_4_F_9_-NDI; for both compounds, all the slurry and recrystallization by evaporation experiments led to the starting material phase Form α, with exception for the slurry experiment in ACN that produced the solvate forms C_3_F_7_-NDI·ACN and C_4_F_9_-NDI·ACN. These solvate forms quickly release the acetonitrile and are converted into very poorly crystalline forms, which are not ascribable to the starting material and are labeled C_3_F_7_-NDI Form γ and C_4_F_9_-NDI Form γ, respectively. These forms are metastable and convert into the starting structure (Form α) over time. C_3_F_7_-NDI·ACN and C_4_F_9_-NDI·ACN small crystals were obtained by precipitation using a gradient temperature with a cooling rate of −0.125 °C/min; the crystals are stable as long as they remain in the mother liquor, but their quality was not good enough for SC-XRD.

### 2.2. Crystal Structure

**Figure 1:**
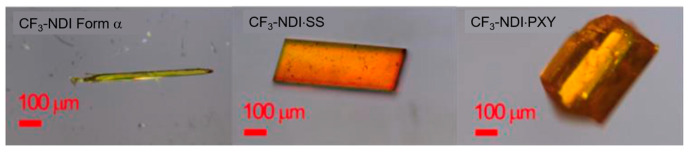
Crystal habits of CF_3_-NDI crystal forms.

Suitable crystals of CF_3_-NDI Form α and CF_3_-NDI·SS were obtained by thermal gradient of ACN and DMSO solutions, respectively, while CF_3_-NDI·PXY crystals were obtained by slow evaporation of the solvent. Crystal structure parameters are listed in Table 1. All the crystals exhibit different crystal habits, as shown in Figure 1: CF_3_-NDI Form α crystalizes as yellow needle-like crystals, CF_3_-NDI·SS grows as orange prism crystals, while CF_3_-NDI·PXY forms dark yellow crystals with a block-like morphology. 

**Figure 2:**
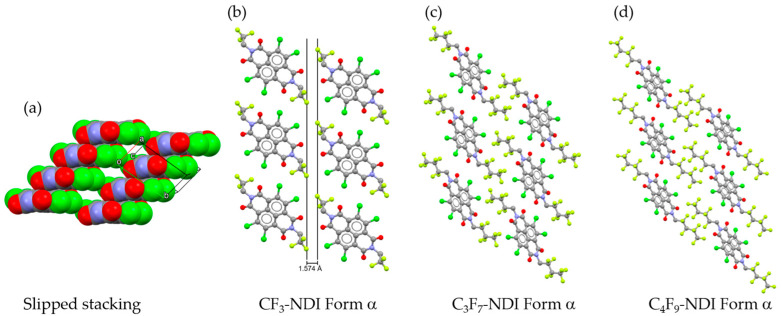
Packing of NDI derivative core along *a* axis (**a**). View of layer separation of CF_3_-NDI Form α (**b**) and interdigitation of the molecules of C_3_F_7_-NDI Form α (**c**) and C_4_F_9_-NDI Form α (**d**).

Both CF_3_-NDI Form α and CF_3_-NDI·PXY crystallize as triclinic with space group P $\bar{1}$ with a half molecule in the asymmetric unit, and in the case of the solvate form, with a half molecule of CF_3_-NDI and a half molecule of paraxylene. The packing of CF_3_-NDI Form α is characterized by layers with slip-stacked molecular packing, observed also in the structure of C_3_F_7_-NDI and C_4_F_9_-NDI Form α (Figure 2a) [4]: the layers are parallel to crystallographic axis a, as shown in Figure 2b. The -CF_3_ groups are not interdigitated, and the two layers show a separation of 1.574 Å, as shown at Figure 2. Despite the presence of the slippery plane, the crystals are brittle upon mechanical deformation [31,32].

**Figure 3:**
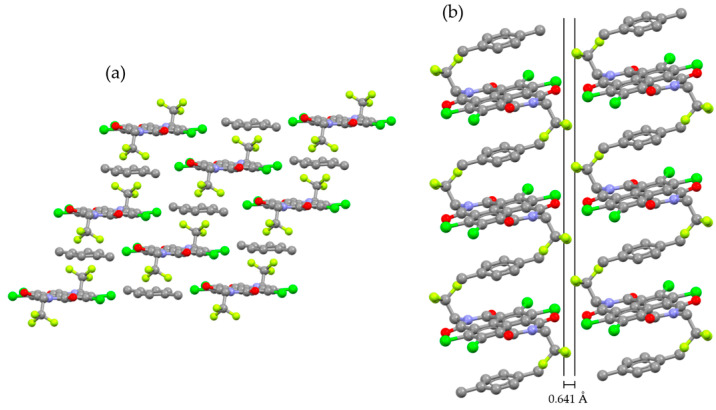
Alternated slip-stack of NDI and PXY molecule (**a**) and view of layer separation of CF_3_-NDI·PXY (**b**).

In CF_3_-NDI·PXY, the solvent molecules are alternated between the NDI molecules keeping the slip-stacked packing, as shown in Figure 3. Similarly, to CF_3_-NDI Form α, CF_3_-NDI·PXY displays a slip plane with a short distance of 0.641 Å between the layers.

**Figure 4:**
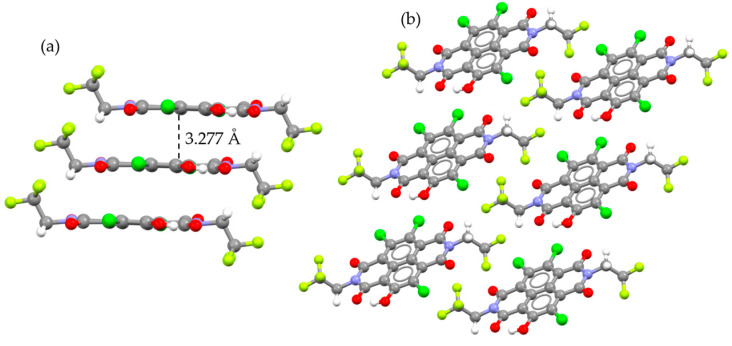
π–π stacking plane distance (**a**) and crystal packing of CF_3_-NDI·SS (**b**); only the CF_3_-NDI-OH are shown, but it should be considered that 20% of the solid solution has only Cl in the core.

CF_3_-NDI·SS crystallizes as triclinic P1, with the inversion center lost due to the substitution of one chlorine atom with a hydroxyl group, which the diffraction data suggest presenting with 80%. The hydroxyl group was confirmed also by the ^13^C-NMR (Figure S2). Interestingly, the crystal structure of CF_3_-NDI·SS shows the shortest distance between π-planes (3.277 Å) (Figure 4) and a flat core with the lowest torsion angle of all the structures, suggesting that if a pure compound is obtained with the same packing parameters, it should give high mobilities in the solid state (Figure S4). Another indicator for expecting high charge-carrier mobilities is the high density of the crystal structure (2.069 g·cm^−3^) [4].

The specific configuration of crystal packing relies, amongst other factors, on the main chain’s characteristics, the presence or absence of side chains, the structural characteristics of the side chains (linear or branched), and their attachment position (regioregularity). Therefore, the solid-state packing of the NDI core is crucial not only for comprehending the functionality of electronic devices utilizing oligomers but also for grasping the similarity of the structures despite the differences in the chains. A way to describe the packing in such a crystal structure is the description of ‘‘pitch” and “roll’’ angles (P and R) and their relative χ and ψ [33]. The estimated values of the P and R angles and the packing parameters of the NDI derivative crystal structures are reported in Table 2. We have compared the χ and ψ values obtained with the ones reported for NDIs with linear alkyl side chains (number of carbon atoms = 1, 2, 4, 5, 6, 8, 10, 12, 14) [34]. Two distinct family groups are identified, one with short chains (*n* = 1–6) and the second with longer chains. Our data fall within a relatively scattered region of the short chains, suggesting that the chain interactions do not significantly impact the crystal packing (Figure S9). Indeed, for short-chain NDI derivatives, there are many interactions at play, and it is difficult to identify a clear pattern.

Regarding the π–π stacking plane distance, both CF_3_-NDI·SS and CF_3_-NDI·PXY show short distances, 3.277 Å and 3.395 Å, which can be associated with the darker orange color of the crystals.

### 2.3. Thermal Characterization

**Figure 5:**
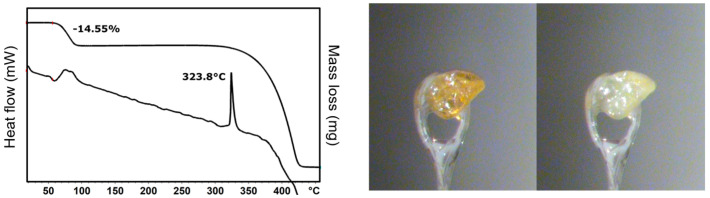
Left, TGA curve of CF_3_-NDI·PXY, which shows the loss of solvent; right, pictures of crystal before (orange crystal) and after (yellow crystal) desolvation.

Thermal analysis (DSC and TGA) on the starting materials and the new obtained forms were performed. DSC of CF_3_-NDI Form α was performed with two cycles of heating and cooling. No transitions were observed, except for the peaks related to the melting at 323.5 °C in the heating cycles and the peak of crystallization during the cooling (Figure S10). Similar behavior was observed for CF_3_-NDI·SS, which shows only the melting peak at 304.0 °C (Figure S11). TGA-EGA analysis of CF_3_-NDI·PXY confirmed the presence and the nature of the solvent. The weight loss (−14.55%) is comparable with the one expected with a stoichiometry 1:1 (15.75%). The slightly lower experimental value is probably due to the instability of the solvate in ambient conditions. The PXY molecules are readily released from the crystal lattice at approximately 63 °C or within a few hours under ambient conditions. The desolvation is easily identified since the crystal color changes from orange to light yellow (Figure 5, right), with a single-crystal-to-single-crystal conversion. The formation of solvates in organic semiconductor materials is relatively uncommon and has been rarely documented in the literature [35,36,37].

**Figure 6:**
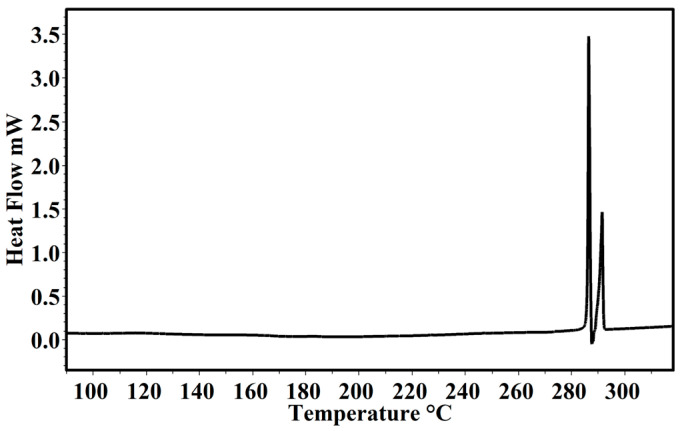
DSC analysis of C_3_F_7_-NDI, showing the presence of melting and crystallization followed by a complete melting, 2 °C/min).

The thermal behavior of the species C_3_F_7_-NDI Form α and C_4_F_9_-NDI Form α were never described previously. The DSC curve of C_3_F_7_-NDI Form α shows a double event just before melting, which corresponds to the melting of Form α at 286 °C followed by recrystallization into Form β, which melts at 291 °C (Figure 6). Upon cooling, the Form β is obtained, since the second heating shows only one endothermic event at 291 °C. To confirm the presence of different forms we characterized the material recovered after differential scanning calorimetry (DSC) analysis of several batches using X-ray powder diffraction (XRPD). The resulting crystal phases were inconsistent across batches, with some yielding pure C_3_F_7_-NDI Form α, while others presented a mixture of Form α and Form β. These findings suggest that C_3_F_7_-NDI initially crystallizes in the metastable Form β, which readily converts to the more stable C_3_F_7_-NDI Form α at room temperature.

**Figure 7:**
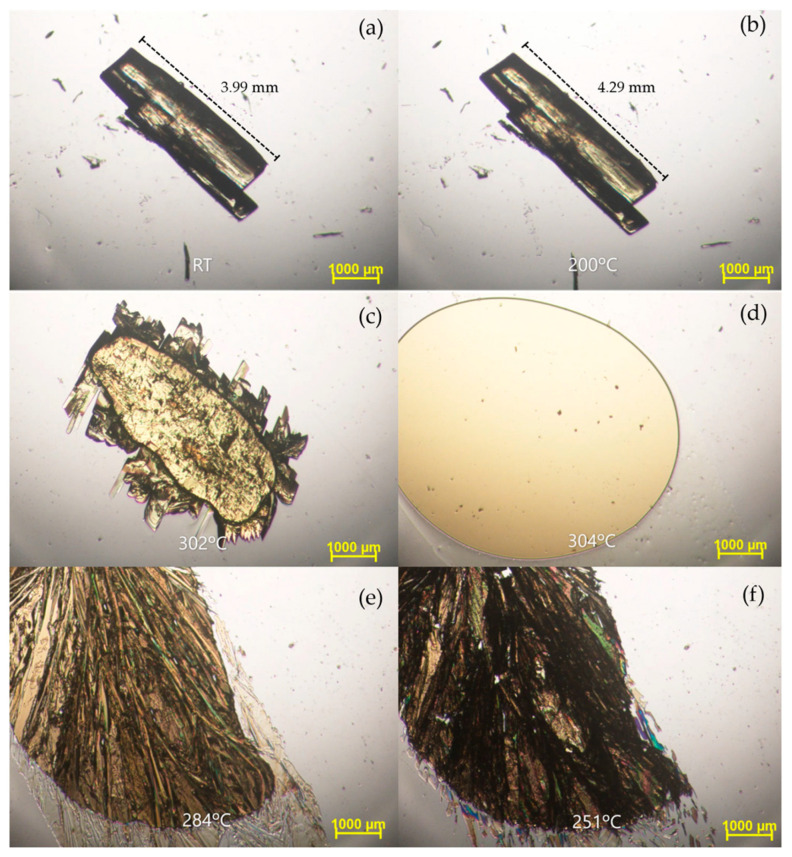
Pictures from hot stage microscopy of C_3_F_7_-NDI: (**a**) initial state of C_3_F_7_-NDI·Form α crystal, (**b**) expanded crystal after thermal expansion and respective dimension, (**c**) melting and crystallization at 302 °C, (**d**) complete melting at 304 °C, (**e**) recrystallization at 284 °C and (**f**) 251 °C.

Hot stage microscopy (HSM) of C_3_F_7_-NDI Form α showed high thermal expansion mainly along the long axis of the crystals, which can be followed under the microscope; upon cooling, the crystals shrink to the starting dimensions (Figure 7).

At slightly higher temperature (302 °C) than the DSC, it is possible to observe the double event of the melting of the C_3_F_7_-NDI Form α and recrystallization into Form β, followed by the complete melting at 304 °C (see Figure 7). In HSM, during the cooling process, the C_3_F_7_-NDI crystallizes at 284 °C as Form β, and at 251 °C a solid–solid transition is observed which is ascribable to the transition C_3_F_7_-NDI Form β → C_3_F_7_-NDI Form α.

**Figure 8:**
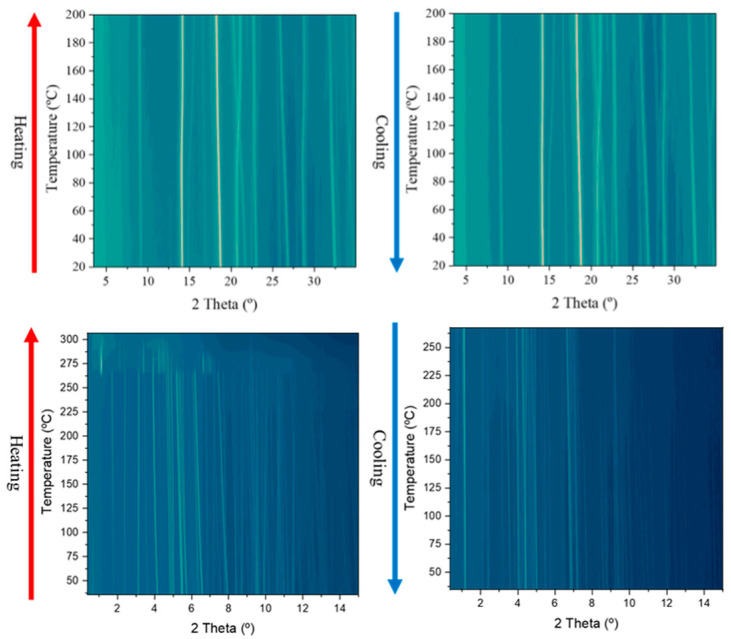
VT-XRPD ofC_3_F_7_-NDI in heating and cooling (**top**), showing the same behavior for both since the thermal expansion of C_3_F_7_-NDI is reversible, and (**bottom**) C_4_F_9_-NDI, which shows the non-reversible transition from C_4_F_9_-NDI Form α to C_4_F_9_-NDI Form β.

To further investigate the thermal expansion behavior of C_3_F_7_-NDI, Variable Temperature X-ray Powder Diffraction (VT-XRPD) was carried out by heating the material to 200 °C and then cooling it down. (Figure 8, top). The XRD patterns during heating revealed shifts in peak positions, indicative of thermal expansion, a phenomenon also observed via hot stage microscopy (HSM). The PASCal software (http://pascal.chem.ox.ac.uk/, accessed on 25 July 2024) was employed to confirm and quantify the thermal expansion coefficients using the cell parameters listed in Table S2. In triclinic systems, the principal axes—along which the material exhibits purely linear thermal responses—are orthogonal but do not correspond to the unit-cell axes. The thermal expansion coefficients were found to be very anisotropic: X_1_ is slightly negative (−11 MK^−1^) and X_3_ shows a colossal positive thermal expansion (PTE) [38] of 320 MK^−1^ with an overall volume expansion of 383 MK^−1^ (for more details see Table S3). This value is much higher than that of common organic systems, which typically present a volume expansion coefficient of 168 MK^−1^ [39,40].

It is noteworthy that negative thermal expansion (NTE) has been observed in other materials, as evidenced by the findings of a CSD study, which indicated that approximately 34% of organic crystals exhibited NTE in at least one orthogonal axis [39]. Furthermore, in the realm of semiconductors, several examples have been documented, such as those observed in BHH-BTBT and NDI-C6 [27,41,42].

In the case of C_4_F_9_-NDI Form α, the DSC curve shows a solid–solid transition around 276 °C ascribed to the transition C_4_F_9_-NDI Form α → C_4_F_9_-NDI Form β, followed by the melting point at 283 °C (Figure S12). Upon cooling, C_4_F_9_-NDI recrystallizes into Form β; the second heating confirms the presence of C_4_F_9_-NDI Form β as only the melting at 283 °C is observed. The transition C_4_F_9_-NDI Form α → C_4_F_9_-NDI Form β was observed also with VT-XRPD, in which it occurs at slightly lower temperature, around 258 °C (Figure 8, bottom).

The VT-XRPD analysis of C_4_F_9_-NDI Form β reveals an intense peak at a low angle, typically associated with the molecular length, as observed for NDI-C_6_ [27]. The presence of a low angle peak ascribable to a long axis suggests that the C_4_F_9_-NDI molecules in Form β pack in a less interdigitated manner, resulting in an increased d-spacing. During the heating process, the VT-XRPD also shows a significant thermal shift in the peak position before the transition occurs, which was quantified using PASCal software. The principal axes’ thermal expansion coefficients and their orientations are summarized in Table S5. Also, in this case, C_4_F_9_-NDI Form α presents a very anisotropic thermal expansion with X_1_, X_2_, and X_3_, respectively, −79, 184, and 287 MK^−1^ and an overall volume thermal expansion coefficient of 398 MK^−1^.

**Figure 9:**
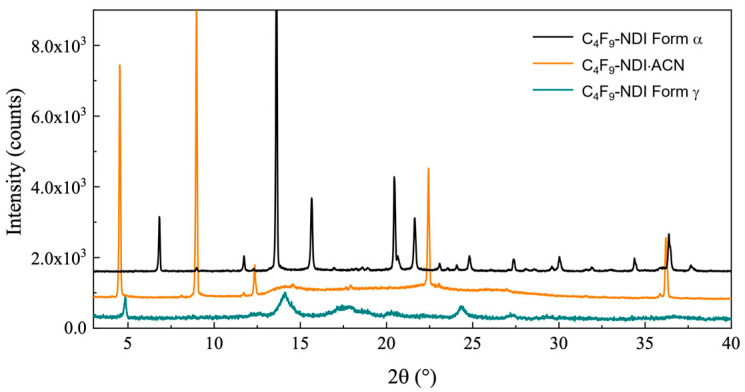
XRPD curve of C_4_F_9_-NDI Form γ transition from C_4_F_9_-NDI Form γ to C_4_F_9_-NDI Form α.

C_4_F_9_-NDI∙ACN crystals, obtained by precipitation using a gradient temperature, are very unstable; for this reason, their XRPD pattern was collected in the presence of some mother liquor to avoid desolvation. Indeed, C_4_F_9_-NDI∙ACN rapidly loses the solvent converting into C_4_F_9_-NDI Form γ. This last form is characterized by a low crystallinity with peaks not ascribable to Form α nor Form β (Figure 9). The release of solvent from C_4_F_9_-NDI∙ACN crystals submerged in fomblin was observed through HSM, clearly showing the formation of bubbles around 78 °C (Figure S13). Unfortunately, the low crystallinity of this phase does not allow further characterization. For that reason, this crystal phase is not appealing for optoelectronic applications.

## 3. Materials and Methods

The CF_3_-NDI, C_3_F_7_-NDI, and C_4_F_9_-NDI were provided by BASF SE (Ludwigshafen am Rhein, Germany) and used without further purification.

### 3.1. Polymorph Screening

The solubility of the three materials was assessed in 14 different solvents at room temperature (see Table S1 in Supplementary Materials). Since the molecules exhibit different solubilities, the solvents used for the recrystallization experiments were chosen according to the solubility. Recrystallization by solvent evaporation at room temperature was carried out in the solvents where the solubility was higher than 5 mg/mL (for CF_3_-NDI, acetonitrile, acetone, DMF, DMSO, ethyl acetate, paraxylene, THF, toluene; for C_3_F_7_-NDI, acetone, dichloromethane, DMF, ethyl acetate, paraxylene, THF, toluene; for C_4_F_9_-NDI, acetone, DMF, ethyl acetate, paraxylene, THF, toluene), and for the remaining solvents slurry experiments at RT were performed.

Slow cooling crystallization was performed using crystallization systems Crystal16 instrument (Technobis, Alkmaar, The Netherlands). Acetonitrile (ACN) solution of CF_3_-NDI with concentration of 12.5 mg/mL was cooled from 75 °C to 25 °C with a cooling rate of 0.125 °C/min; when the solvent was DMSO, the solution with a concentration of 33.3 mg/mL was cooled with the same rate from 100 °C to 25 °C. In the case of C_3_F_7_-NDI and C_4_F_9_-NDI, slow cooling recrystallization was performed in ACN solution with a concentration of 3 mg/mL from 75 °C to 25 °C and a cooling rate of 0.125 °C/min. All the crystals were left to dry in room conditions before XRD characterization.

During the polymorph screening, slurry experiments of CF_3_-NDI were performed in isopropanol (2PR), chloroform (CHF), dichloromethane (DCM), ethanol (ETH), water (H_2_O), and methanol (MET), and the solid residue was measured by XRD after one day, three days and ten days. In addition, anti-solvent precipitation and precipitation by gradient temperature were performed in ACN. In all cases, CF_3_-NDI Form α was obtained.

### 3.2. X-ray Powder Diffraction (XRPD)

The powders obtained by recrystallization and the starting material of all the compounds were characterized by XRPD to identify the crystalline phases. The XRD patterns were obtained in Bragg−Brentano geometry, over the 2θ range of 3–40°, with a step size of 0.01° and a speed of 10.0°/min, using a Rigaku (Tokyo, Japan) MiniFlex 600 diffractometer with Cu Kα (λ = 1.54178 Å) radiation generated from a copper sealed tube with 40 kV and 15 mA.

### 3.3. Variable Temperature X-ray Powder Diffraction (VT-XRPD)

VT-XRPD in transmission mode was performed at ALBA Synchrotron light facility (Barcelona, Spain) at BL04-MSPD (Material Science and Powder Diffraction) beamline using glass capillaries with a diameter of 0.5 mm, spinning during the measurement. CF_3_-NDI and C_4_F_9_-NDI powders were filled in 0.5 mm glass capillaries. The beamline was equipped with the high-throughput Position Sensitive Detector (PSD) MYTHEN (Baden, Switzerland) and an FMB Oxford (Oxford, UK) hot air blower to control the temperature [43]. The data were collected with a beam energy of 30 keV (0.41235(1) Å) [44]. The characterization of C_4_F_9_-NDI was executed in a temperature range from 20 °C to 275 °C in continuous heating with a 5 °C/min heating rate, while for CF_3_-NDI, only room temperature powder pattern was collected.

VT-XRPD of C_3_F_7_-NDI was performed in reflection mode on a PANalytical X’Pert Pro (Almelo, The Netherlands) automated diffractometer with an X’Celerator detector in Bragg–Brentano geometry over the 2θ range of 3–30°, under continuous scan mode with a step size of 0.0167°, using Cu Ka radiation (λ = 1.5418 Å) without a monochromator with 40 mA and 40 kV. The diffractometer was equipped with an Anton Paar (Graz, Austria) TTK 450 system for measurements at a controlled temperature.

### 3.4. Single-Crystal X-ray Diffraction (SCXRD)

Data collection for single-crystal X-ray diffraction of CF_3_-NDI·PXY, obtained from slow evaporation at room temperature of a p-xylene solution, was performed with a Rigaku (Tokyo, Japan) Oxford Diffraction Xcalibur diffractometer with a Sapphire 3 detector using Mo Kα radiation (λ = 0.71073 Å) at room temperature.

A crystal of CF_3_-NDI·SS, obtained from DMSO by slow evaporation at room temperature, was mounted on a cryo-loop and used for a low-temperature (160 K) X-ray structure determination. All measurements were performed on a Rigaku (Tokyo, Japan) Oxford Diffraction XtaLAB Synergy diffractometer with a HyPix 6000HE hybrid pixel array detector using Mo Kα radiation (λ = 0.71073 Å) from a PhotonJet micro-focus X-ray source and an Oxford Cryosystems Cryostream 800 + cooler (Oxford, UK). A crystal of CF_3_-NDI Form α, obtained from acetonitrile by slow-cooling crystallization, was mounted following the description above, but in this case the radiation used was Cu Kα (λ = 1.54184 Å).

The crystal structure was solved using OLEX 2-1.5 [45] software by recurring SHELXT codes that use intrinsic phasing and refined with SHELXL-2019/2 with least square L.S. command [46].

Crystallography data are summarized in Table 1. CCDC 2373442, 2373443, and 2373444 contain the supplementary crystallographic data for this paper. The data can be obtained free of charge from the Cambridge Crystallographic Data Centre via https://www.ccdc.cam.ac.uk/structures/?, accessed on 25 July 2024.

For visualization and image acquisition of the crystal structures, CCDC Mercury 2022.1.0 [47,48] was used.

### 3.5. Differential Scanning Calorimetry (DSC)

The starting materials and the crystals obtained were characterized by differential scanning calorimetry (DSC) using a Mettler-Toledo (Columbus, OH, USA) DSC-1 instrument. The samples were prepared by weighing approximately 1–4 mg of the powder or crystals in aluminum pans (40 μL) and then sealed with an aluminum cover. Before the measurement, the pan cover was pierced, and the measurements were performed with a pinhole during two cycles of heating and cooling with a rate of 5 °C/min under a dry N_2_ atmosphere (flow rate 80 mL/min). Different rates of heating and cooling were tried for better clarity of the thermal events, namely 2 °C/min, 5 °C/min, 10 °C/min, and 20 °C/min. All the data were analyzed using STARe software (https://www.mt.com/us/en/home/products/Laboratory_Analytics_Browse/TA_Family_Browse/TA_software_browse.html, accessed on 25 July 2024).

### 3.6. Thermo-Gravimetric Analysis/Evolved Gas Analysis (TGA-EGA)

To characterize the solvate forms of the different materials, TGA-EGA was performed on Mettler-Toledo (Columbus, OH, USA) TGA coupled with a ThermoFisher (Waltham, MA, USA) Nicolet iS 10IR FT_IR spectrometer, with a scan rate of 10 °C/min and analyzed using STARe software.

### 3.7. Hot Stage Microscopy (HSM)

The thermal behavior of the different crystals was studied by hot stage microscopy (HSM); the influence of the temperature was observed on single crystals of C_3_F_7_-NDI∙ACN and C_4_F_9_-NDI∙ACN, obtained by precipitation by gradient temperature in ACN with a cooling rate of −0.125 °C/min. The samples were prepared by placing the crystals on a glass slide and covering with a coverslip. The sample was then positioned inside of the sealed heating chamber. The thermal behavior was analyzed using an OLYMPUS (Tokyo, Japan) BX41 stereomicroscope equipped with a LINKAM (Salfords, UK) LTS350 platinum plate for temperature control and VISICAM analyzer. During the experiment, time-lapse images were taken using a NIKON (Tokyo, Japan) DS FI3 high-speed camera and analyzed using Nikon NIS Elements software (https://www.microscope.healthcare.nikon.com/en_EU/products/software/nis-elements, accessed on 25 July 2024) and Linksys32.

### 3.8. Attenuated Total Reflection/Fourier-Transform Infrared (ATR-FTIR) Spectroscopy

Infrared spectroscopy was performed on all the crystal forms of CF_3_-NDI, which includes starting material Form I, solid solution, and the solvate phase. The spectra were obtained using a Fourier-transform infrared spectrometer: ThermoFisher, (Waltham, MA, USA) Nicolet iS50 and Detector: DTGS ATR, Perkin Elmer, (Waltham, MA, USA). The spectra were measured over the range of 4000–400 cm^−1^.

### 3.9. Nuclear Magnetic Resonance (NMR)

^13^C-NMR (100 MHz) and ^1^H-NMR (400 MHz) were performed on a sample of CF_3_-NDI∙SS using a Bruker (Billerica, MA, USA) Avance Neo 600 MHz spectrometer equipped with cryoprobe PRODIGY and cooled with liquid nitrogen.

### 3.10. PASCal

PASCal—Principal Axis Strain Calculator is a web-based tool used to determine the principal coefficients of thermal expansion from variable-temperature lattice parameters [49]. The tool requires the temperature and the unit cell parameter values at each temperature as input data. For C_3_F_7_-NDI, we used the data from the VT-XRD obtained using a PANalytical (Almelo, The Netherlands) X’Pert Pro, while in the case of C_4_F_9_-NDI, the diffractograms acquired at ALBA were used. In both cases, the cell parameters were refined using Pawley refinement. The tool returns as output the principal axis of thermal expansion and the orientation of the principal axes relative to the axes of the unit cell.

## 4. Conclusions

We studied the polymorphic behavior of three core-chlorinated NDIs with fluoroalkyl-chain compounds CF_3_-NDI, C_3_F_7_-NDI, and C_4_F_9_-NDI, which are appealing for the field of organic electronics, and, in particular, C_3_F_7_-NDI and C_4_F_9_-NDI are proved to have satisfactory electrical behavior. In the case of CF_3_-NDI, we did not find any evidence of different polymorphs; only one pure crystal phase was observed, but we isolated a p-xylene solvate form and serendipitously a solid solution was also discovered and their structures solved. The solid solution was obtained by an unexpected partial decomposition of the original compound in DMSO during the recrystallization process. The crystal structure of CF_3_-NDI was compared with the known crystal structures of C_3_F_7_-NDI and C_4_F_9_-NDI. Even though all the structures show a slip-stacked molecular packing, the NDI cores are characterized by quite different pitch and roll angle values. Interestingly, CF_3_-NDI Form α shows a slip plane of 1.574 Å between layers, but the crystals are brittle. CF_3_-NDI·SS shows a packing very different from CF_3_-NDI Form α, with a short distance between π-planes (3.277 Å) and interdigitated chains; however, it was not possible to obtain this crystal forms with only CF_3_-NDI. It is noteworthy that in CF_3_-NDI·PXY, solvent molecules engage in donor–acceptor interactions, suggesting the potential for the formation of co-crystals. The polymorph screening carried out for C_3_F_7_-NDI and C_4_F_9_-NDI gave rise to three new crystal forms for each compound. Both compounds present an ACN solvate very unstable outside the mother liquor which rapidly converts into a poorly crystalline form with main peaks not ascribable to the other polymorphs, namely C_3_F_7_-NDI Form γ and C_4_F_9_-NDI Form γ. Furthermore, the thermal characterization showed the presence of high-temperature polymorphs called, respectively, C_3_F_7_-NDI Form β and C_4_F_9_-NDI Form β, which are metastable at room temperature and convert back into Form α. C_3_F_7_-NDI presents a big thermal expansion upon heating that can even be visualized by the change in dimensions of the crystal by HSM, at a volume coefficient expansion of 383MK^−1^ quantified by Pascal. Also, C_4_F_9_-NDI Form α is characterized by high thermal expansion, which should be considered during the preparation of the devices.

## Figures and Tables

**Table 1:** Crystal structure parameters of CF_3_-NDI Form α, CF_3_-NDI·SS, CF_3_-NDI·PXY, C_3_F_7_-NDI Form α, and C_4_F_9_-NDI Form α.

	CF_3_-NDI Form α	CF_3_-NDI·SS	CF_3_-NDI·PXY	C_3_F_7_-NDI Form α	C_4_F_9_-NDI Form α
CCDC number	2373444	2373442	2373443	BUYJIP [4]	BUYJEL [4]
Temperature (K)	160.15	160.15	RT	100	100
Formula	C_18_H_4_Cl_4_F_6_N_2_O_4_	C_18_H_4.8_Cl_3.2_F_6_N_2_O_4.8_	C_18_H_4_Cl_4_F_6_N_2_O_4_, C_8_H_10_	C_22_H_4_Cl_4_F_14_N_2_O_4_	C_24_H_4_Cl_4_F_18_N_2_O_4_
Molecular weight	568.03	553.46	674.19	768.07	868.08
Crystal system	Triclinic	Triclinic	Triclinic	Triclinic	Triclinic
Space group	$P\bar{1}$	P1	$P\bar{1}$	$P\bar{1}$	$P\bar{1}$
a (Å)	4.8722(2)	5.83510(10)	7.7798(7)	5.1039(19)	5.5055(7)
b (Å)	8.8027(3)	8.09990(10)	8.1543(9)	10.358(4)	9.8095(11)
c (Å)	11.5608(2)	9.7935(2)	10.6415(7)	12.395(5)	12.7073(15)
α (°)	75.075(2)	97.9620(10)	91.60(7)	111.077(19)	87.786(6)
β (°)	89.777(2)	95.3400(10)	101.96(7)	90.07(2)	81.088(7)
γ (°)	82.792(3)	102.323(2)	90.58(8)	96.67(2)	87.289(7)
$V\;({Å}^{3}$)	475.103	444.242	660.08	606.645	676.888
Z/Z’	1/0.5	1/1	1/0.5	1/0.5	1/0.5
Density (g·cm^−3^)	1.985	2.069	1.696	2.102	2.13
F(000)	280	274	338		
GOF on F^2^	1.156	1.100	1.039	1.076	1.030
R_1_ (on F, I > 2σ(I)/R_ex_)	0.0306	0.0695	0.0477	0.0918	0.0397
WR_2_ (F^2^ all data) R_wp_	0.0856	0.2323	0.1049	0.2512	0.1027

**Table 2:** Pitch and roll angles and π–π distances for NDI derivatives. More details on values’ determination are reported in the Supplementary Materials.

Compound	Χ (deg)	Ψ (deg)	Stacking Vector (Å)	d (π-π) (Å)	P (deg)	R (deg)
CF_3_-NDI	74.42	49.37	4.872	3.449	20.78	42.61
C_3_F_7_-NDI	69.89	48.44	5.104	3.463	26.87	44.36
C_4_F_9_-NDI	78.39	40.88	5.506	3.438	17.86	50.45
CF_3_-NDI·SS	75.08	38.39	5.835	3.316	24.37	54.06
CF_3_-NDI	74.42	49.37	4.872	3.449	20.78	42.61

## Data Availability

NMR and FTIR spectra, DSC curves and pitch and roll calculations are reported in the supporting information. The original contributions presented in the study are included in the article/supplementary material, further inquiries can be directed to the corresponding author/s.

## Supplementary Materials

The following supporting information can be downloaded at: https://www.mdpi.com/article/10.3390/molecules29184376/s1, Table S1. Summary of solubility assessment of CF_3_-NDI, C_3_F_7_-NDI, and C_4_F_9_-NDI; Figure S1. H-NMR of CF_3_-NDI·SS; Figure S2. C-NMR of CF_3_-NDI·SS; Figure S3. FTIR of CF_3_-NDI Form α in red and CF_3_-NDI·SS in green; Figure S4. NDI core with labeled atoms and dihedral angles of all the different crystal forms of CF_3_-NDI; Figure S5. O--H interactions of Form α (thermodynamic stable form) of each molecule; Figure S6. Intermolecular potentials of CF_3_-NDI Form α, C_3_F_7_-NDI Form α and C_4_F_9_-NDI Form α; Figure S7. Schematic representation of the stacking vector (SV), angle χ and ψ between two parallel NDI cores; Figure S8. Visual representation of pitch and roll angles for CF_3_-NDI crystal packing; Figure S9. Clustering of the different χ and ψ values; Figure S10. DSC curves of CF_3_-NDI Form α; Figure S11. DSC curves of CF3-NDI·SS; Table S2. Unit-cell parameters used as input for the PASCal calculation, obtained by Pawley refinement of the reported cell of C_3_F_7_-NDI Form α at different temperatures. Collected with PANalytical X’Pert Pro; Table S3. Values (α_X_) of the principal axes of thermal expansion (X_1_, X_2_, and X_3_) and their orientation in regards of the cell axis, a, b and c of C_3_F_7_-NDI Form α; Table S4. Unit cell parameters used as input for the PASCal calculation, obtained by Pawley refinement at different temperatures of the reported cell of C_4_F_9_-NDI Form α. Collected at ALBA Synchrotron light facility (Barcelona); Table S5. Values (α_X_) of the principal axis of thermal expansion (X1, X2, and X3) and their orientation in regards of the cell axis, a, b, and c of C4F9-NDI Form α; Figure S12. DSC of C_4_F_9_-NDI Form α which shows the non-reversible transition to C4F9-NDI Form β; Figure S13. (a) Desolvation of C_3_F_7_-NDI·ACN and (b) C4F9-NDI·ACN.
