# Supplementary material for: Polymorph Screening of Core-Chlorinated Naphthalene Diimides with Different Fluoroalkyl Side-Chain Lengths
Source: Molecules. 2024 Sep 14;29(18):4376. doi: 10.3390/molecules29184376 (PMC11434339; doi:10.3390/molecules29184376)

checkCIF/PLATON report

Structure factors have been supplied for datablock(s) ofet-5-i_cu

THIS REPORT IS FOR GUIDANCE ONLY. IF USED AS PART OF A REVIEW PROCEDURE FOR PUBLICATION, IT SHOULD NOT REPLACE THE EXPERTISE OF AN EXPERIENCED CRYSTALLOGRAPHIC REFEREE.

No syntax errors found.   CIF dictionary   Interpreting this report

Datablock: ofet-5-i_cu

```
Bond precision:   C-C = 0.0023 A          Wavelength=1.54184

Cell:           a=4.8722(2)        b=8.8027(3)       c=11.5608(2)
                alpha=75.075(2)    beta=89.777(2)    gamma=82.792(3)
Temperature:    160 K

                 Calculated                    Reported
Volume           475.10(3)                     475.10(3)
Space group      P -1                          P -1
Hall group       -P 1                          -P 1
Moiety formula   C18 H4 Cl4 F6 N2 O4           C18 H4 Cl4 F6 N2 O4
Sum formula      C18 H4 Cl4 F6 N2 O4           C18 H4 Cl4 F6 N2 O4
Mr               568.03                        568.03
Dx,g cm-3        1.985                         1.985
Z                1                             1
Mu (mm-1)        6.552                         6.552
F000             280.0                         280.0
F000'            282.47
h,k,lmax         6,11,14                       6,11,14
Nref             2058                          2002
Tmin,Tmax        0.767,0.769                   0.265,1.000
Tmin'            0.162

Correction method= # Reported T Limits: Tmin=0.265 Tmax=1.000
AbsCorr = GAUSSIAN

Data completeness= 0.973           Theta(max)= 79.413

R(reflections)= 0.0306( 1940)                     wR2(reflections)=
                                                  0.0860( 2002)
S = 1.156                    Npar= 163
```

The following ALERTS were generated. Each ALERT has the format
test-name_ALERT_alert-type_alert-level.
Click on the hyperlinks for more details of the test.

Alert level B

```
PLAT031_ALERT_4_B Refined Extinction Parameter Within Range of ...      1.714 Sigma
PLAT934_ALERT_3_B Number of (Iobs-Icalc)/Sigma(W) > 10 Outliers ..          2 Check
```

Alert level C

```
ABSTY02_ALERT_1_C  An _exptl_absorpt_correction_type has been given without
            a literature citation. This should be contained in the
            _exptl_absorpt_process_details field.
            Absorption correction given as gaussian
PLAT911_ALERT_3_C Missing FCF Refl Between Thmin & STh/L=    0.600          3 Report
```

Alert level G

```
PLAT242_ALERT_2_G Low    'MainMol' Ueq as Compared to Neighbors of         C9 Check
PLAT432_ALERT_2_G Short Inter X...Y Contact  O1       ..C6       .       2.93 Ang.
                                                   1+x,y,z  =      1_655 Check
PLAT434_ALERT_2_G Short Inter HL..HL Contact Cl1      ..F1       .       3.12 Ang.
                                               2-x,2-y,1-z  =      2_776 Check
PLAT883_ALERT_1_G No Info/Value for _atom_sites_solution_primary .     Please Do !
PLAT912_ALERT_4_G Missing # of FCF Reflections Above STh/L=  0.600         54 Note
PLAT941_ALERT_3_G Average HKL Measurement Multiplicity ..........         4.7 Low
PLAT978_ALERT_2_G Number C-C Bonds with Positive Residual Density.          8 Info
PLAT992_ALERT_5_G Repd & Actual _reflns_number_gt Values Differ by          2 Check
```

0 **ALERT level A** = Most likely a serious problem - resolve or explain
2 **ALERT level B** = A potentially serious problem, consider carefully
2 **ALERT level C** = Check. Ensure it is not caused by an omission or oversight
8 **ALERT level G** = General information/check it is not something unexpected

2 ALERT type 1 CIF construction/syntax error, inconsistent or missing data
4 ALERT type 2 Indicator that the structure model may be wrong or deficient
3 ALERT type 3 Indicator that the structure quality may be low
2 ALERT type 4 Improvement, methodology, query or suggestion
1 ALERT type 5 Informative message, check

It is advisable to attempt to resolve as many as possible of the alerts in all categories. Often the minor alerts point to easily fixed oversights, errors and omissions in your CIF or refinement strategy, so attention to these fine details can be worthwhile. In order to resolve some of the more serious problems it may be necessary to carry out additional measurements or structure refinements. However, the purpose of your study may justify the reported deviations and the more serious of these should normally be commented upon in the discussion or experimental section of a paper or in the "special_details" fields of the CIF. checkCIF was carefully designed to identify outliers and unusual parameters, but every test has its limitations and alerts that are not important in a particular case may appear. Conversely, the absence of alerts does not guarantee there are no aspects of the results needing attention. It is up to the individual to critically assess their own results and, if necessary, seek expert advice.

Publication of your CIF in IUCr journals

A basic structural check has been run on your CIF. These basic checks will be run on all CIFs submitted for publication in IUCr journals (*Acta Crystallographica*, *Journal of Applied Crystallography*, *Journal of Synchrotron Radiation*); however, if you intend to submit to *Acta Crystallographica Section C* or *E* or *IUCrData*, you should make sure that full publication checks are run on the final version of your CIF prior to submission.

Publication of your CIF in other journals

Please refer to the *Notes for Authors* of the relevant journal for any special instructions relating to CIF submission.

PLATON version of 12/09/2022; check.def file version of 09/08/2022

Datablock ofet-5-i_cu - ellipsoid plot

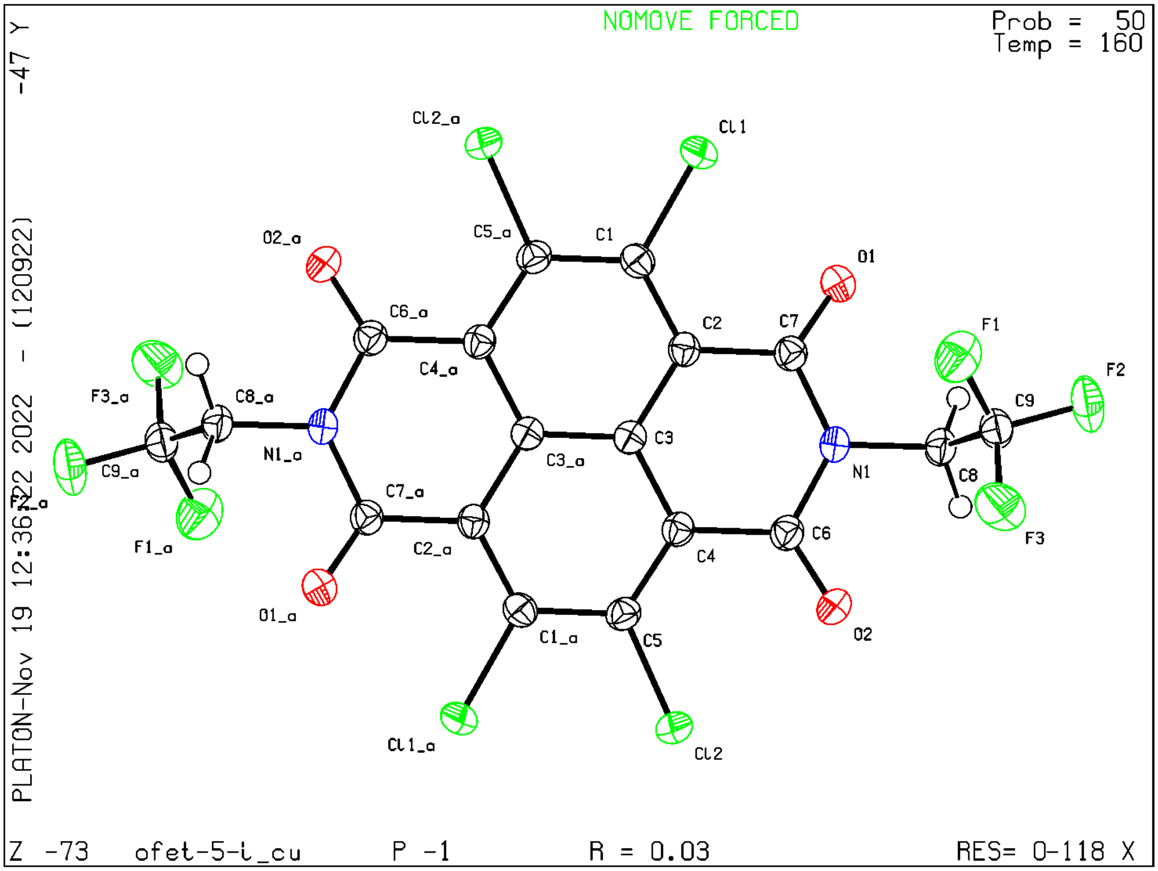

Supplement: Supplementary file 1 [file molecules-29-04376-s001.zip › checkcif CF3-NDI-I-alfa.pdf]
