# Supplementary material for: Polymorph Screening of Core-Chlorinated Naphthalene Diimides with Different Fluoroalkyl Side-Chain Lengths
Source: Molecules. 2024 Sep 14;29(18):4376. doi: 10.3390/molecules29184376 (PMC11434339; doi:10.3390/molecules29184376)

checkCIF/PLATON report

Structure factors have been supplied for datablock(s) ofet5_pxy_datared

THIS REPORT IS FOR GUIDANCE ONLY. IF USED AS PART OF A REVIEW PROCEDURE FOR PUBLICATION, IT SHOULD NOT REPLACE THE EXPERTISE OF AN EXPERIENCED CRYSTALLOGRAPHIC REFEREE.

No syntax errors found. CIF dictionary Interpreting this report

Datablock: ofet5_pxy_datared

Bond precision: C-C = 0.0047 A Wavelength=0.71073

Cell:	a=7.7798(7)	b=8.1543(9)	c=10.6415(7)
	alpha=91.595(7)	beta=101.963(7)	gamma=90.584(8)
Temperature:	293 K		

	Calculated	Reported
Volume	660.08(11)	660.08(10)
Space group	P -1	P -1
Hall group	-P 1	-P 1
Moiety formula	C18 H4 Cl4 F6 N2 O4, C8 H10	C18 H4 Cl4 F6 N2 O4, C8 H10
Sum formula	C26 H14 Cl4 F6 N2 O4	C26 H14 Cl4 F6 N2 O4
Mr	674.19	674.19
Dx,g cm-3	1.696	1.696
Z	1	1
Mu (mm-1)	0.530	0.530
F000	338.0	338.0
F000'	338.80	
h,k,lmax	10,11,14	9,10,13
Nref	3677	2672
Tmin,Tmax		0.818,1.000
Tmin'		

Correction method= # Reported T Limits: Tmin=0.818 Tmax=1.000
AbsCorr = MULTI-SCAN

Data completeness= 0.727 Theta(max)= 29.461

R(reflections)= 0.0477(1890) wR2(reflections)= 0.1227(2672)

S = 1.039 Npar= 210

The following ALERTS were generated. Each ALERT has the format
test-name_ALERT_alert-type_alert-level.
Click on the hyperlinks for more details of the test.

Alert level A

```
PLAT029_ALERT_3_A _diffrn_measured_fraction_theta_full value Low .      0.910 Why?
```

Alert level B

```
PLAT911_ALERT_3_B Missing FCF Refl Between Thmin & STh/L=    0.600        218 Report
```

Alert level C

```
ABSTY02_ALERT_1_C  An _exptl_absorpt_correction_type has been given without
            a literature citation. This should be contained in the
            _exptl_absorpt_process_details field.
            Absorption correction given as multi-scan
PLAT053_ALERT_1_C Minimum Crystal Dimension Missing (or Error) ...     Please Check
PLAT054_ALERT_1_C Medium  Crystal Dimension Missing (or Error) ...     Please Check
PLAT055_ALERT_1_C Maximum Crystal Dimension Missing (or Error) ...     Please Check
PLAT213_ALERT_2_C Atom O2              has ADP max/min Ratio .....        3.4 prolat
PLAT234_ALERT_4_C Large Hirshfeld Difference O2       --C6       .       0.16 Ang.
PLAT244_ALERT_4_C Low    'Solvent' Ueq as Compared to Neighbors of        C10 Check
PLAT250_ALERT_2_C Large U3/U1 Ratio for Average U(i,j) Tensor ....        2.1 Note
PLAT260_ALERT_2_C Large Average Ueq of Residue Including       C10      0.116 Check
PLAT334_ALERT_2_C Small <C-C> Benzene Dist.   C10      -C11_b    .       1.37 Ang.
PLAT340_ALERT_3_C Low Bond Precision on  C-C Bonds ..............     0.00469 Ang.
PLAT906_ALERT_3_C Large K Value in the Analysis of Variance ......      3.187 Check
PLAT910_ALERT_3_C Missing # of FCF Reflection(s) Below Theta(Min).          6 Note
```

Alert level G

```
PLAT199_ALERT_1_G Reported _cell_measurement_temperature ..... (K)        293 Check
PLAT200_ALERT_1_G Reported   _diffrn_ambient_temperature ..... (K)        293 Check
PLAT242_ALERT_2_G Low    'MainMol' Ueq as Compared to Neighbors of         C9 Check
PLAT300_ALERT_4_G Atom Site Occupancy of O1A        Constrained at       0.75 Check
PLAT300_ALERT_4_G Atom Site Occupancy of O2A        Constrained at       0.75 Check
PLAT300_ALERT_4_G Atom Site Occupancy of O1         Constrained at       0.25 Check
PLAT300_ALERT_4_G Atom Site Occupancy of O2         Constrained at       0.25 Check
PLAT301_ALERT_3_G Main Residue  Disorder ..............(Resd  1  )        12% Note
PLAT802_ALERT_4_G CIF Input Record(s) with more than 80 Characters          1 Info
PLAT883_ALERT_1_G No Info/Value for _atom_sites_solution_primary .     Please Do !
PLAT912_ALERT_4_G Missing # of FCF Reflections Above STh/L=  0.600        645 Note
PLAT913_ALERT_3_G Missing # of Very Strong Reflections in FCF ....          2 Note
PLAT941_ALERT_3_G Average HKL Measurement Multiplicity ...........        1.6 Low
PLAT978_ALERT_2_G Number C-C Bonds with Positive Residual Density.          5 Info
```

1 **ALERT level A** = Most likely a serious problem - resolve or explain
1 **ALERT level B** = A potentially serious problem, consider carefully
13 **ALERT level C** = Check. Ensure it is not caused by an omission or oversight
14 **ALERT level G** = General information/check it is not something unexpected

7 ALERT type 1 CIF construction/syntax error, inconsistent or missing data

```
 6 ALERT type 2 Indicator that the structure model may be wrong or deficient
 8 ALERT type 3 Indicator that the structure quality may be low
 8 ALERT type 4 Improvement, methodology, query or suggestion
 0 ALERT type 5 Informative message, check
```

It is advisable to attempt to resolve as many as possible of the alerts in all categories. Often the minor alerts point to easily fixed oversights, errors and omissions in your CIF or refinement strategy, so attention to these fine details can be worthwhile. In order to resolve some of the more serious problems it may be necessary to carry out additional measurements or structure refinements. However, the purpose of your study may justify the reported deviations and the more serious of these should normally be commented upon in the discussion or experimental section of a paper or in the "special_details" fields of the CIF. checkCIF was carefully designed to identify outliers and unusual parameters, but every test has its limitations and alerts that are not important in a particular case may appear. Conversely, the absence of alerts does not guarantee there are no aspects of the results needing attention. It is up to the individual to critically assess their own results and, if necessary, seek expert advice.

Publication of your CIF in IUCr journals

A basic structural check has been run on your CIF. These basic checks will be run on all CIFs submitted for publication in IUCr journals (*Acta Crystallographica*, *Journal of Applied Crystallography*, *Journal of Synchrotron Radiation*); however, if you intend to submit to *Acta Crystallographica Section C* or *E* or *IUCrData*, you should make sure that full publication checks are run on the final version of your CIF prior to submission.

Publication of your CIF in other journals

Please refer to the *Notes for Authors* of the relevant journal for any special instructions relating to CIF submission.

PLATON version of 12/09/2022; check.def file version of 09/08/2022

Datablock ofet5_pxy_datared - ellipsoid plot

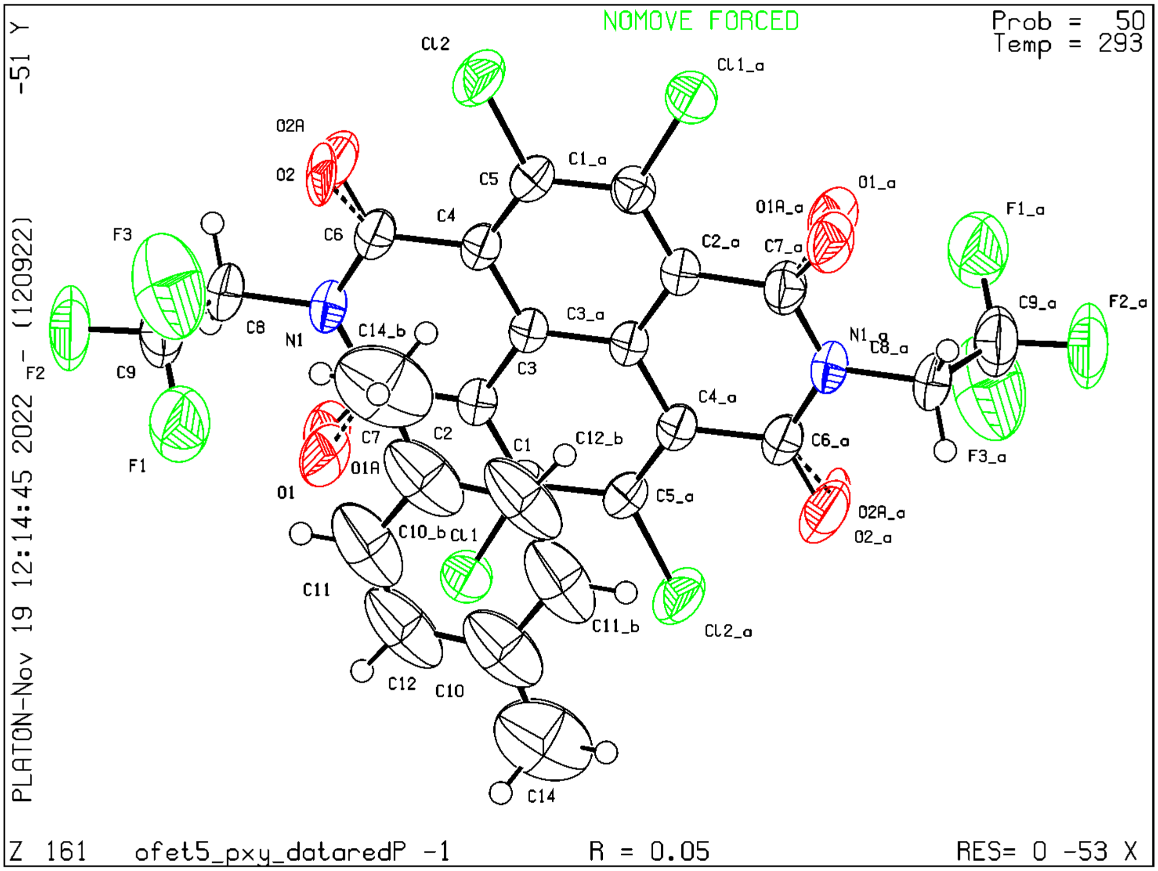

Supplement: Supplementary file 1 [file molecules-29-04376-s001.zip › checkcif CF3-NDI-PXY.pdf]
