# Supplementary material for: Polymorph Screening of Core-Chlorinated Naphthalene Diimides with Different Fluoroalkyl Side-Chain Lengths
Source: Molecules. 2024 Sep 14;29(18):4376. doi: 10.3390/molecules29184376 (PMC11434339; doi:10.3390/molecules29184376)

checkCIF/PLATON report

Structure factors have been supplied for datablock(s) ofet5_o_free

THIS REPORT IS FOR GUIDANCE ONLY. IF USED AS PART OF A REVIEW PROCEDURE FOR PUBLICATION, IT SHOULD NOT REPLACE THE EXPERTISE OF AN EXPERIENCED CRYSTALLOGRAPHIC REFEREE.

No syntax errors found. CIF dictionary Interpreting this report

Datablock: ofet5_o_free

Bond precision: C-C = 0.0075 A Wavelength=0.71073

Cell:	a=5.8351(1)	b=8.0999(1)	c=9.7935(2)
	alpha=97.962(1)	beta=95.340(1)	gamma=102.323(2)
Temperature:	160 K		

	Calculated	Reported
Volume	444.242(14)	444.242(14)
Space group	P 1	P 1
Hall group	P 1	P 1
Moiety formula	C18 H4.79 Cl3.21 F6 N2 O4.79	C18 H4.788 Cl3.212 F6 N2 O4.788
Sum formula	C18 H4.79 Cl3.21 F6 N2 O4.79	C18 H4.79 Cl3.21 F6 N2 O4.79
Mr	553.46	553.46
Dx,g cm-3	2.069	2.069
Z	1	1
Mu (mm-1)	0.651	0.651
F000	273.7	274.0
F000’	274.35	
h,k,lmax	8,11,14	8,11,14
Nref	5818[2909]	4571
Tmin,Tmax	0.861,0.925	0.714,1.000
Tmin’	0.861	

Correction method= # Reported T Limits: Tmin=0.714 Tmax=1.000
AbsCorr = GAUSSIAN

Data completeness= 1.57/0.79 Theta(max)= 31.300

R(reflections)= 0.0695(4387) wR2(reflections)= 0.2361(4571)

S = 1.100 Npar= 319

The following ALERTS were generated. Each ALERT has the format
test-name_ALERT_alert-type_alert-level.
Click on the hyperlinks for more details of the test.

Alert level B

```
PLAT230_ALERT_2_B Hirshfeld Test Diff for    Cl2      --C5       .       11.0 s.u.
PLAT230_ALERT_2_B Hirshfeld Test Diff for    Cl4      --C10      .       17.0 s.u.
PLAT934_ALERT_3_B Number of (Iobs-Icalc)/Sigma(W) > 10 Outliers ..          2 Check
```

Alert level C

```
ABSTY02_ALERT_1_C  An _exptl_absorpt_correction_type has been given without
            a literature citation. This should be contained in the
            _exptl_absorpt_process_details field.
            Absorption correction given as gaussian
DIFMN02_ALERT_2_C  The minimum difference density is < -0.1*ZMAX*0.75
            _refine_diff_density_min given =     -1.351
            Test value =     -1.275
DIFMN03_ALERT_1_C  The minimum difference density is < -0.1*ZMAX*0.75
            The relevant atom site should be identified.
PLAT042_ALERT_1_C Calc. and Reported MoietyFormula Strings  Differ     Please Check
PLAT077_ALERT_4_C Unitcell Contains Non-integer Number of Atoms ..     Please Check
PLAT098_ALERT_2_C Large Reported Min.  (Negative) Residual Density      -1.35 eA-3
PLAT340_ALERT_3_C Low Bond Precision on  C-C Bonds ..............     0.00747 Ang.
PLAT790_ALERT_4_C Centre of Gravity not Within Unit Cell: Resd.  #          1 Note
              C18 H4.79 Cl3.21 F6 N2 O4.79
PLAT915_ALERT_3_C No Flack x Check Done: Low Friedel Pair Coverage         70 %
PLAT918_ALERT_3_C Reflection(s) with I(obs) much Smaller I(calc) .          2 Check
PLAT939_ALERT_3_C Large Value of Not (SHELXL) Weight Optimized S .      10.20 Check
```

Alert level G

```
PLAT002_ALERT_2_G Number of Distance or Angle Restraints on AtSite          3 Note
PLAT007_ALERT_5_G Number of Unrefined Donor-H Atoms .............          1 Report
PLAT068_ALERT_1_G Reported F000 Differs from Calcd (or Missing)...     Please Check
PLAT111_ALERT_2_G ADDSYM Detects New (Pseudo) Centre of Symmetry .         96 %Fit
PLAT113_ALERT_2_G ADDSYM Suggests Possible Pseudo/New  Space Group       P-1 Check
              Check Model Parameter Symmmetry for Reflection Data Support
PLAT172_ALERT_4_G The CIF-Embedded .res File Contains DFIX Records          2 Report
PLAT242_ALERT_2_G Low    'MainMol' Ueq as Compared to Neighbors of         C9 Check
PLAT242_ALERT_2_G Low    'MainMol' Ueq as Compared to Neighbors of        C18 Check
PLAT301_ALERT_3_G Main Residue  Disorder ..............(Resd  1  )         3% Note
PLAT304_ALERT_4_G Non-Integer Number of Atoms in ..... (Resd  1  )      38.79 Check
PLAT432_ALERT_2_G Short Inter X...Y Contact  Cl1       ..C6       .       3.24 Ang.
                                               1+x,y,z  =      1_655 Check
PLAT432_ALERT_2_G Short Inter X...Y Contact  Cl1       ..C16      .       3.25 Ang.
                                              x,-1+y,z  =      1_545 Check
PLAT434_ALERT_2_G Short Inter HL..HL Contact Cl2       ..F6       .       3.01 Ang.
                                            -1+x,y,1+z  =      1_456 Check
PLAT434_ALERT_2_G Short Inter HL..HL Contact Cl3       ..Cl1      .       2.69 Ang.
```

```
                                                -1+x,1+y,z  =      1_465 Check
PLAT434_ALERT_2_G Short Inter HL..HL Contact Cl4      ..F2       .       3.04 Ang.
                                                 1+x,y,-1+z  =      1_654 Check
PLAT860_ALERT_3_G Number of Least-Squares Restraints ............          5 Note
PLAT883_ALERT_1_G No Info/Value for _atom_sites_solution_primary .     Please Do !
PLAT912_ALERT_4_G Missing # of FCF Reflections Above STh/L=  0.600        361 Note
PLAT941_ALERT_3_G Average HKL Measurement Multiplicity ..........        4.4 Low
PLAT978_ALERT_2_G Number C-C Bonds with Positive Residual Density.          3 Info
```

---

  0 **ALERT level A** = Most likely a serious problem - resolve or explain
  3 **ALERT level B** = A potentially serious problem, consider carefully
 11 **ALERT level C** = Check. Ensure it is not caused by an omission or oversight
 20 **ALERT level G** = General information/check it is not something unexpected

  5 ALERT type 1 CIF construction/syntax error, inconsistent or missing data
 15 ALERT type 2 Indicator that the structure model may be wrong or deficient
  8 ALERT type 3 Indicator that the structure quality may be low
  5 ALERT type 4 Improvement, methodology, query or suggestion
  1 ALERT type 5 Informative message, check

It is advisable to attempt to resolve as many as possible of the alerts in all categories. Often the minor alerts point to easily fixed oversights, errors and omissions in your CIF or refinement strategy, so attention to these fine details can be worthwhile. In order to resolve some of the more serious problems it may be necessary to carry out additional measurements or structure refinements. However, the purpose of your study may justify the reported deviations and the more serious of these should normally be commented upon in the discussion or experimental section of a paper or in the "special_details" fields of the CIF. checkCIF was carefully designed to identify outliers and unusual parameters, but every test has its limitations and alerts that are not important in a particular case may appear. Conversely, the absence of alerts does not guarantee there are no aspects of the results needing attention. It is up to the individual to critically assess their own results and, if necessary, seek expert advice.

Publication of your CIF in IUCr journals

A basic structural check has been run on your CIF. These basic checks will be run on all CIFs submitted for publication in IUCr journals (*Acta Crystallographica*, *Journal of Applied Crystallography*, *Journal of Synchrotron Radiation*); however, if you intend to submit to *Acta Crystallographica Section C* or *E* or *IUCrData*, you should make sure that full publication checks are run on the final version of your CIF prior to submission.

Publication of your CIF in other journals

Please refer to the *Notes for Authors* of the relevant journal for any special instructions relating to CIF submission.

PLATON version of 12/09/2022; check.def file version of 09/08/2022

Datablock ofet5_o_free - ellipsoid plot

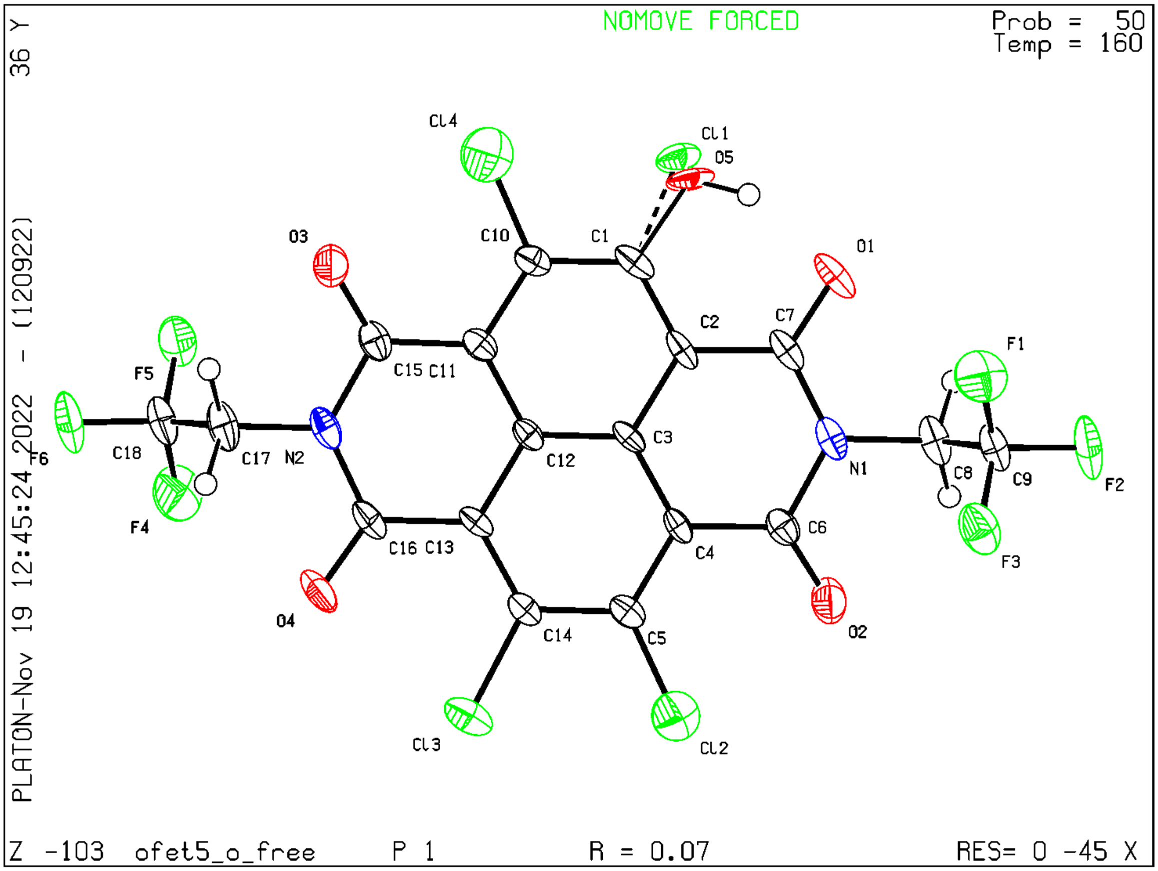

Supplement: Supplementary file 1 [file molecules-29-04376-s001.zip › checkcif CF3-NDI-SS.pdf]
