# Supplementary material for: Polymorph Screening of Core-Chlorinated Naphthalene Diimides with Different Fluoroalkyl Side-Chain Lengths
Source: Molecules. 2024 Sep 14;29(18):4376. doi: 10.3390/molecules29184376 (PMC11434339; doi:10.3390/molecules29184376)
Supplement: Supplementary file 1 [file molecules-29-04376-s001.zip › SI_Polymorph screening of core-chlorinated naphthalene diimides.pdf]

Supporting Materials

Polymorph Screening of Core-Chlorinated Naphthalene Diimides with Different Fluoroalkyl Side-Chain Lengths

Inês de Oliveira Martins [1,2], Marianna Marchini [1,*], Lucia Maini [1,*] and Enrico Modena [2]

[1] Department of Chemistry "Giacomo Ciamician", University of Bologna, Via Selmi 2, 40126 Bologna, Italy; ines.deoliveira2@unibo.it
[2] PolyCrystalLine SPA, Via Della Cooperazione 29, 40059 Medicina, Italy; enrico.modena@polycrystalline.it
[*] Correspondence: marianna.marchini2@unibo.it (M.M.); l.maini@unibo.it (L.M.)

Sommario

Table S1. Summary of solubility assessment of CF_3-NDI, C_3F_7-NDI and C_4F_9-NDI. 2

Figure S1. H-NMR of CF_3-NDI·SS. 3

Figure S2. C-NMR of CF_3-NDI·SS. 4

Figure S3. FTIR of CF_3-NDI Form α in red and CF_3-NDI·SS in green. 4

Figure S4. NDI core with labelled atoms and dihedral angles of all the different crystal forms of CF_3-NDI: .. 5

Figure S5. O--H interactions of Form α (thermodynamic stable form) of each molecule. 5

Figure S6. Intermolecular potentials of CF_3-NDI Form α, C_3F_7-NDI Form α and C_4F_9-NDI Form α. 6

Figure S7. Schematic representation of the stacking vector (SV), angle χ and ψ between two parallel NDI cores. 7

Figure S8. Visual representation of pitch and roll angles for CF_3-NDI crystal packing. 7

Figure S9. Clustering of the different ψ and χ values 7

Figure S10. DSC curves of CF_3-NDI Form α 8

Figure S11. DSC curves of CF_3-NDI·SS 8

Table S2. Unit-cell parameters used as input for the PASCal calculation, obtained by Pawley refinement of the reported cell of C_3F_7-NDI Form α at different temperatures. Collected with PANalytical X'Pert Pro. 8

Table S3. Values (α_X) of the principal axis of thermal expansion (X_1, X_2 and X_3) and their orientation in regards of the cell axis, a, b and c of C_3F_7-NDI Form α. 9

Table S4. Unit-cell parameters used as input for the PASCal calculation, obtained by Pawley refinement at different temperatures of the reported cell of C_4F_9-NDI Form α. Collected at ALBA Synchrotron light facility (Barcelona). 9

Table S5. Values (α_X) of the principal axis of thermal expansion (X_1, X_2 and X_3) and their orientation in regards of the cell axis, a, b and c of C_4F_9-NDI Form α. 11

Figure S12. DSC of C_4F_9-NDI Form α which shows the non-reversible transition to C_4F_9-NDI Form β. 11

Figure S13. a) Desolvation of C_3F_7-NDI·ACN and b) C_4F_9-NDI·ACN. 11

Table S1. Summary of solubility assessment of CF_3-NDI, C_3F_7-NDI and C_4F_9-NDI.

			Solubility (mg/mL)		
**S. No**	**Solvent**	**Abbreviation**	**CF_3-NDI**	**C_3F_7-NDI**	**C_4F_9-NDI**
1	Isopropanol	2PR	< 5	< 5	< 5
2	Acetonitrile	ACN	5	< 5	< 5
3	Acetone	ACT	10	20	5
4	Chloroform	CHF	< 5	< 5	< 5
5	Dichloromethane	DCM	< 5	5	< 5
6	Dimethylformamide	DMF	50	20	5
7	Dimethyl sulfoxide	DMSO	20	< 5	< 5
8	Ethyl acetate	ETA	5	10	5
9	Ethanol	ETH	< 5	< 5	< 5
10	Water	H2O	< 5	< 5	< 5
11	Methanol	MET	< 5	< 5	< 5
12	P-xylene	PXY	10	10	5
13	Tetrahydrofuran	THF	50	50	20
14	Toluene	TOL	10	10	5

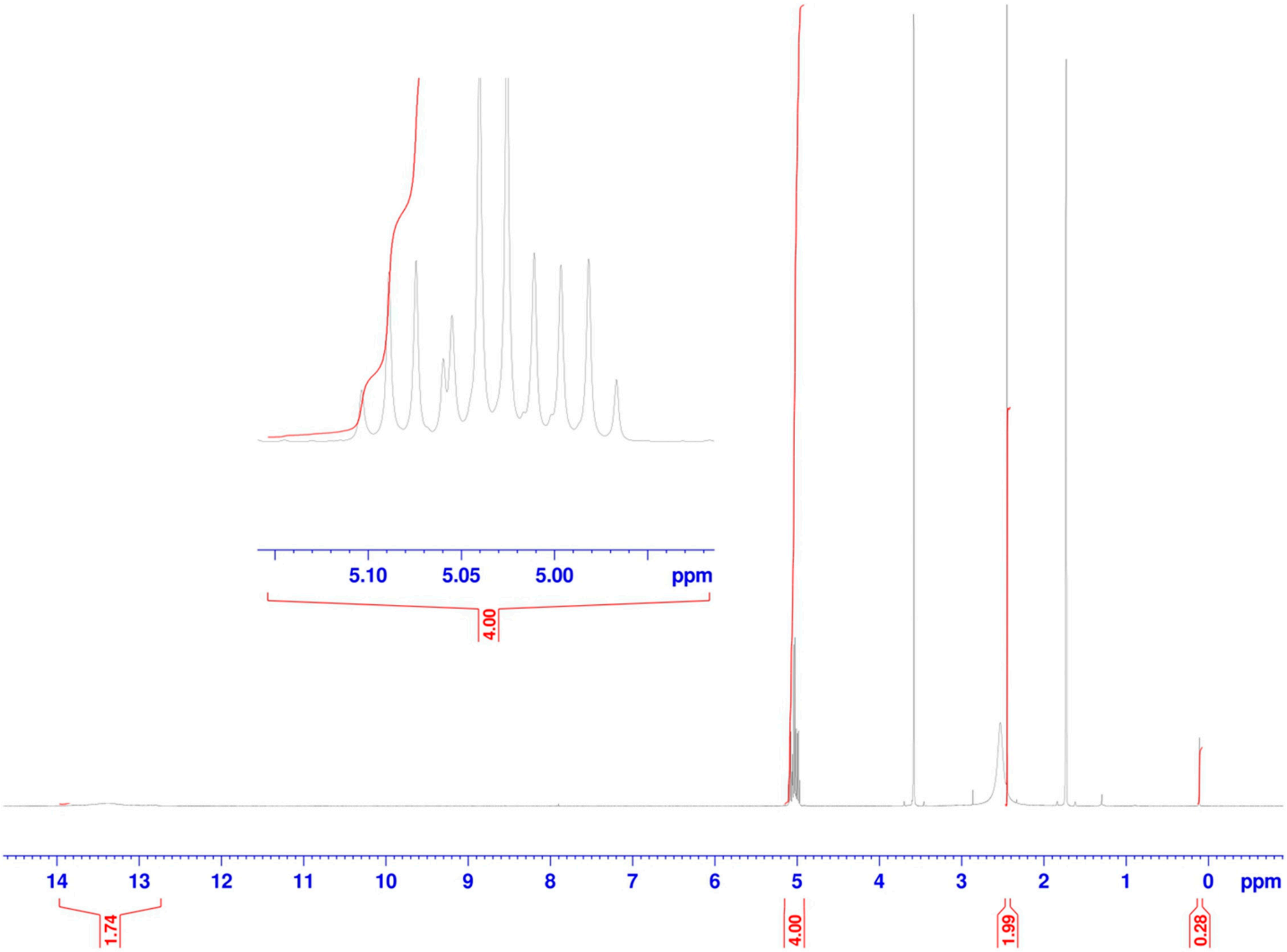

Figure S1. ^{1}H-NMR of CF_3-NDI·SS.

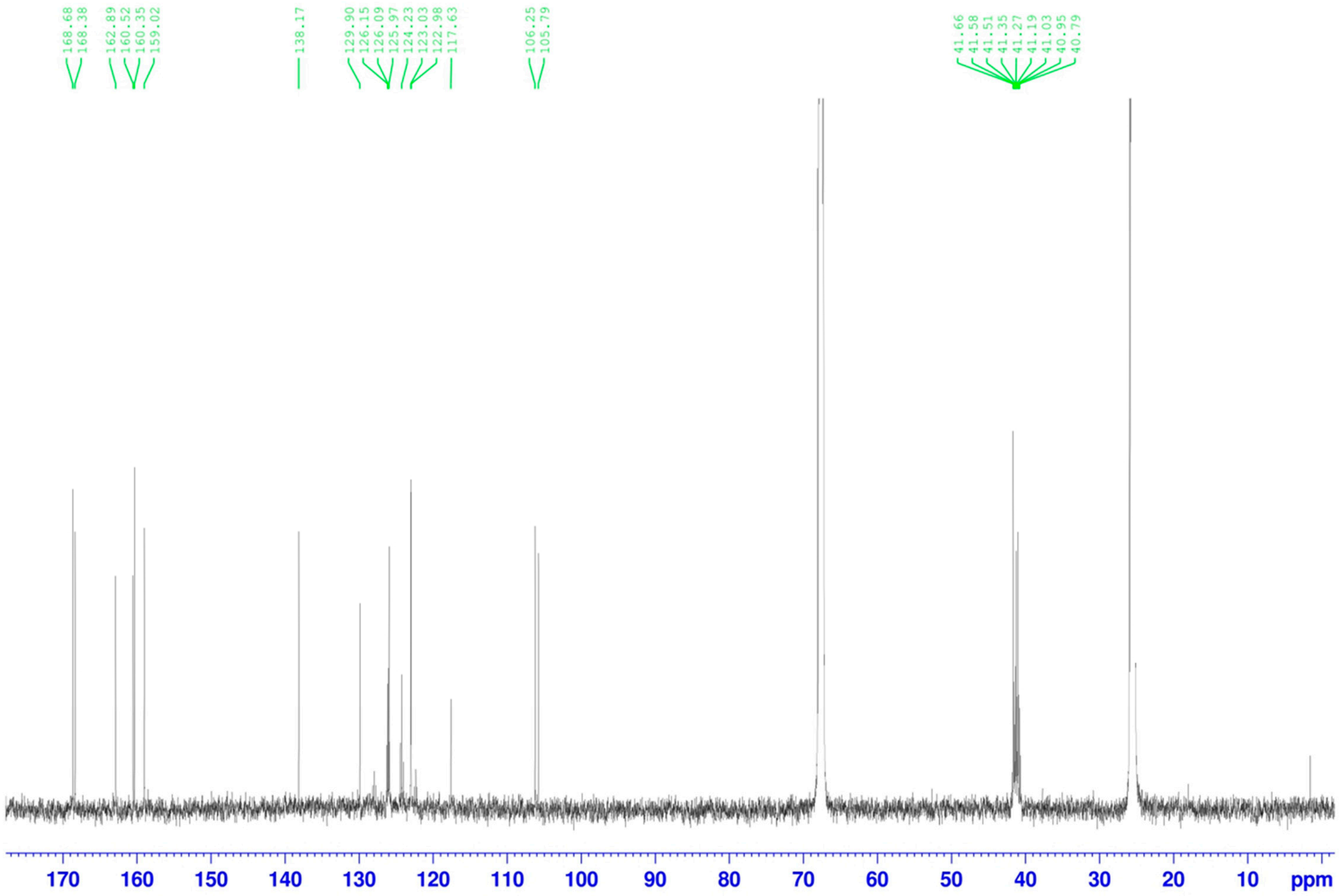

Figure S2. ^{13}C-NMR of CF_3-NDI·SS.

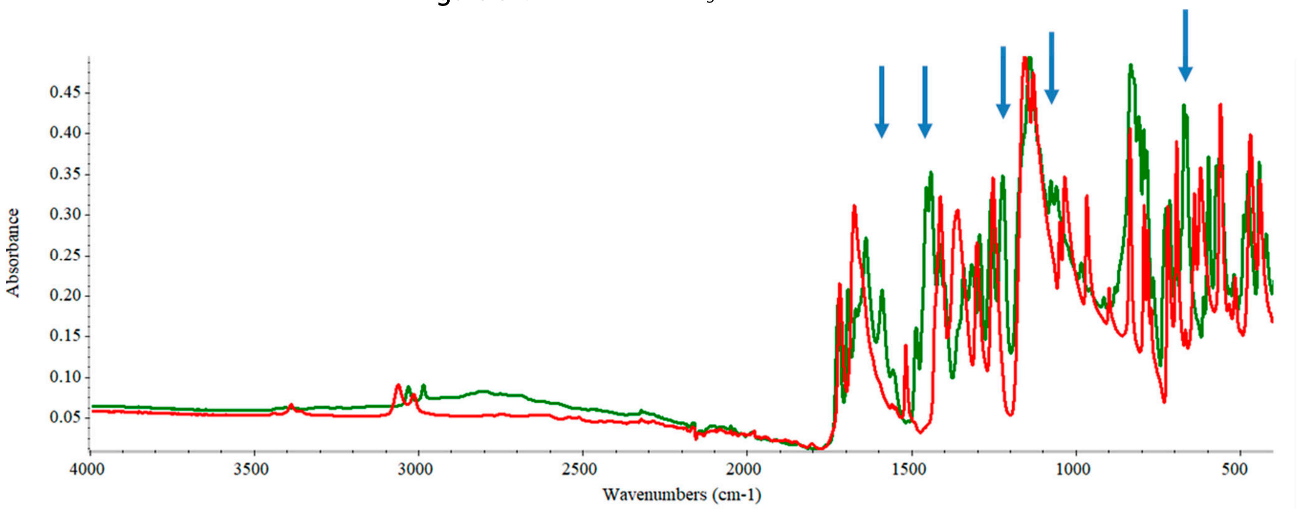

Figure S3. FTIR of CF_3-NDI Form α in red and CF_3-NDI·SS in green.

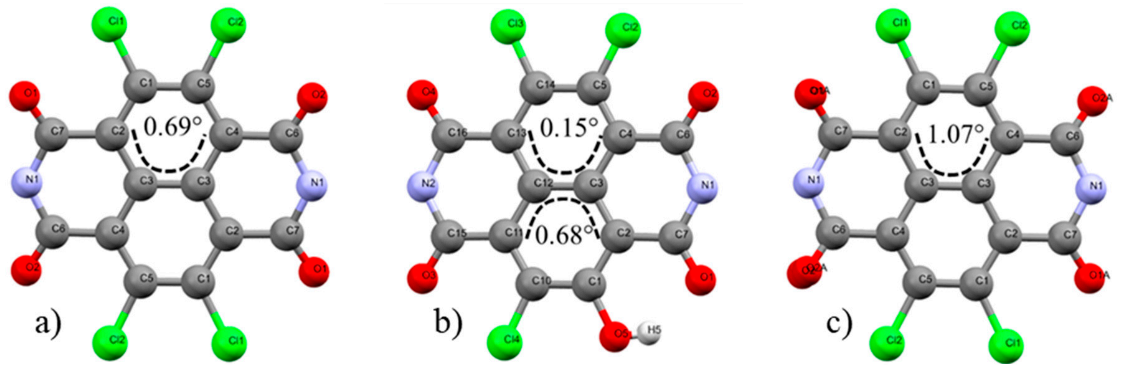

Figure S4. NDI core with labelled atoms and dihedral angles of all the different crystal forms of CF_3-NDI: a) CF_3-NDI Form α, b) CF_3-NDI·SS with only displayed the core of the CF_3-NDI-OH molecule for better clarity and c) CF_3-NDI·PXY.

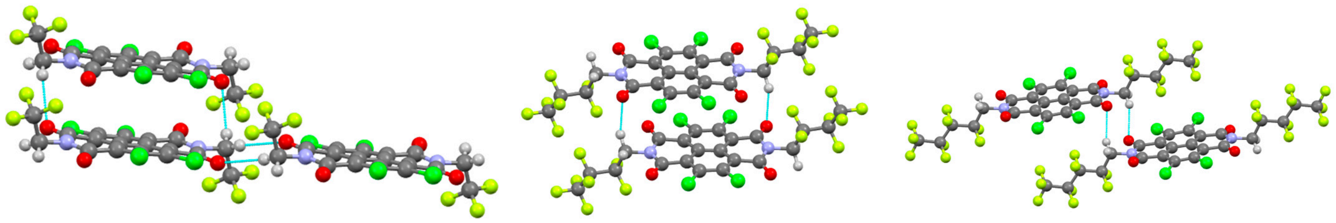

CF_3-NDI Form α | C_3F_7-NDI Form α | C_4F_9-NDI Form α

Figure S5. O--H interactions of Form α (thermodynamic stable form) of each molecule.

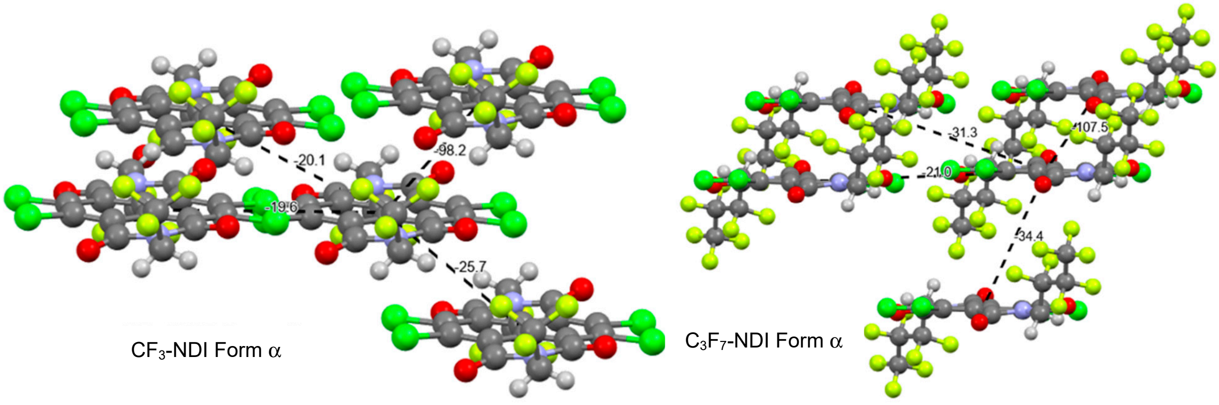

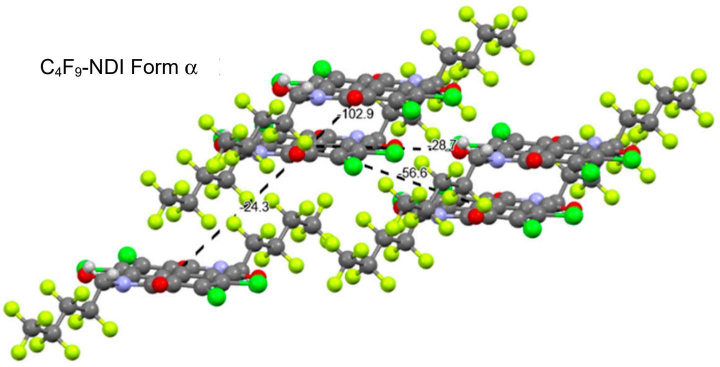

Figure S6. Intermolecular potentials of CF_3-NDI Form α, C_3F_7-NDI Form α and C_4F_9-NDI Form α

Figure S7 shows the definitions of the pitch and roll angles. Let the x-y plane be parallel to the molecular plane of a molecule in a π-stack: the axes x and y represent the long and short axis of the molecule, respectively. The stacking vector (SV) is the distance between the two centroids in two parallel planes. χ and ψ are the angle formed by SV vector with x axes and SV vector with y axes, respectively.

$$\Delta x = SV \cdot \sin(90 - \chi)$$

$$\Delta y = SV \cdot \sin(90 - \psi)$$

$$\tan P = \frac{\Delta x}{d\,(\pi - \pi)}$$

$$\tan R = \frac{\Delta y}{d\,(\pi - \pi)}$$

$$P = \tan^{-1}\frac{\Delta x}{d\,(\pi - \pi)} = \tan^{-1}\frac{SV \cdot \sin(90 - \chi)}{d\,(\pi - \pi)}$$

$$R = \tan^{-1}\frac{\Delta y}{d\,(\pi - \pi)} = \tan^{-1}\frac{SV \cdot \sin(90 - \psi)}{d\,(\pi - \pi)}$$

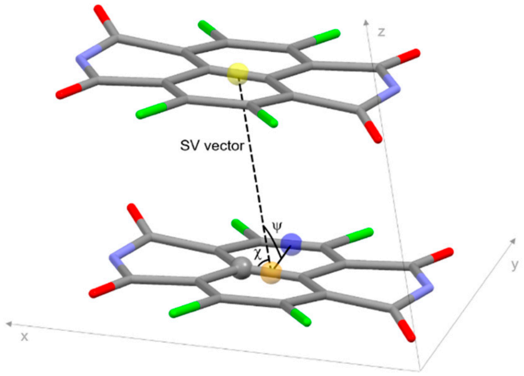

Figure S7. Schematic representation of the stacking vector (SV), angle χ and ψ between two parallel NDI cores.

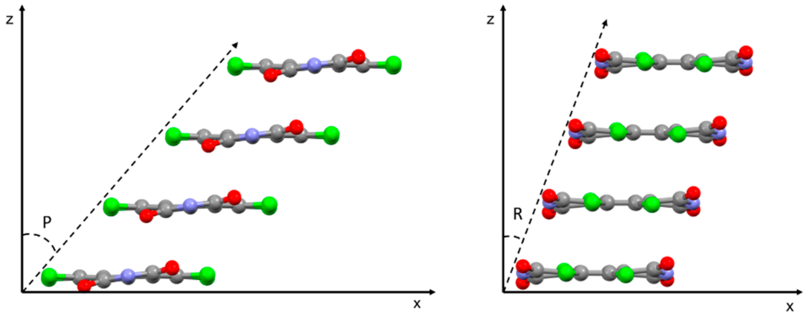

Figure S8. Visual representation of pitch and roll angles for CF_3-NDI crystal packing.

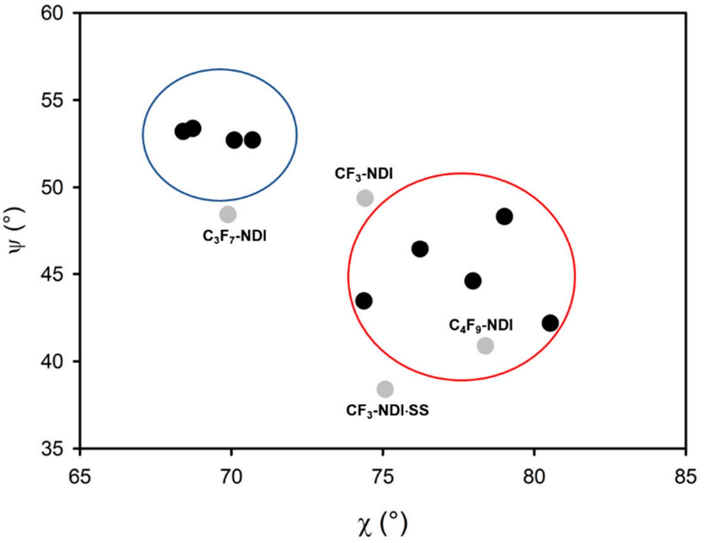

Figure S9. Clustering of the different ψ and χ values calculated for CF_3-NDI, CF_3-NDI·SS, C_3F_7-NDI and C_4F_9-NDI (grey dots), compared with the value estimated by Milita et al. [34] (black dots); the upper dots, for

which ψ ≈ 53°, belong to the long chain NDI derivatives (x = 8, 10, 12, 14). In the red circle are short chain derivatives (x = 1, 2, 4, 5, 6).

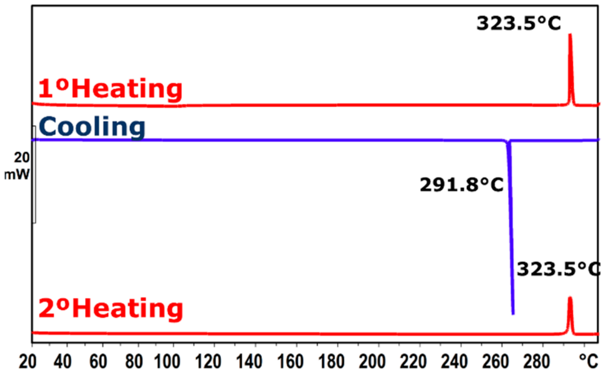

Figure S10. DSC curves of CF_3-NDI Form α, first heating (red line), cooling (blue line) and second heating (red line).

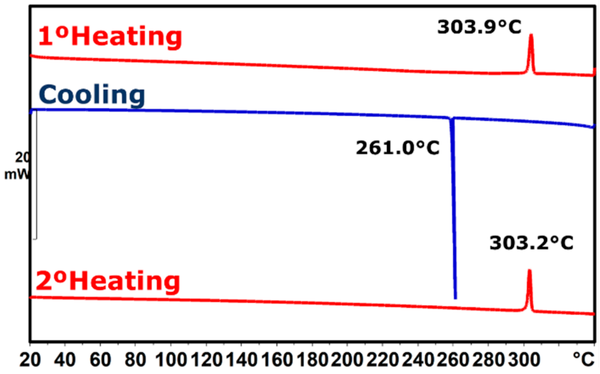

Figure S11. DSC curves of CF_3-NDI·SS, first heating (red line), cooling (blue line) and second heating (red line).

Table S2. Unit-cell parameters used as input for the PASCal calculation, obtained by Pawley refinement of the reported cell of C_3F_7-NDI Form α at different temperatures. Collected with PANalytical X'Pert Pro.

Temperature (°C)	a (Å)	b (Å)	c (Å)	α (°)	β (°)	γ (°)
20	5.144	10.493	12.270	112.12	89.83	97.04
30	5.148	10.506	12.296	112.17	89.89	97.04
40	5.152	10.519	12.326	112.22	89.97	97.03
50	5.156	10.525	12.354	112.25	90.01	97.06
60	5.161	10.523	12.390	112.27	90.03	97.10
70	5.164	10.506	12.460	112.36	89.97	97.00

80	5.169	10.511	12.502	112.45	90.00	96.99
90	5.172	10.530	12.529	112.57	90.11	96.98

Table S3. Values (α_X) of the principal axis of thermal expansion (X_1, X_2 and X_3) and their orientation in regards of the cell axis, a, b and c of C_3F_7-NDI Form α.

	Principal axis	α_X (MK^{-1})	a	b	c
Form α	X_1	-11	0.7595	0.6002	0.2508
	X_2	72	-0.9483	0.3169	-0.0179
	X_3	320	-0.5319	0.0234	0.8465
	Volume	383			

Table S4. Unit-cell parameters used as input for the PASCal calculation, obtained by Pawley refinement at different temperatures of the reported cell of C_4F_9-NDI Form α. Collected at ALBA Synchrotron light facility (Barcelona).

Temperature (°C)	a (Å)	b (Å)	c (Å)	α (°)	β (°)	γ (°)
35.6	5.550	9.932	13.279	84.51	99.43	93.42
38.8	5.551	9.933	13.292	84.44	99.43	93.43
40.3	5.554	9.933	13.312	84.35	99.44	93.47
47.3	5.556	9.935	13.328	84.24	99.49	93.50
51.2	5.557	9.938	13.347	84.14	99.49	93.53
50.5	5.559	9.940	13.359	84.07	99.51	93.55
57.6	5.560	9.943	13.380	83.95	99.52	93.56
59.4	5.562	9.944	13.391	83.90	99.53	93.57
60.2	5.563	9.944	13.404	83.83	99.54	93.59
66.0	5.566	9.946	13.423	83.73	99.55	93.61
72.4	5.568	9.947	13.440	83.64	99.56	93.64
72.3	5.570	9.948	13.451	83.58	99.56	93.66
73.6	5.571	9.948	13.463	83.52	99.56	93.66
77.4	5.574	9.949	13.480	83.44	99.57	93.69
83.1	5.577	9.950	13.499	83.35	99.58	93.71
89.2	5.580	9.950	13.514	83.28	99.58	93.73
92.9	5.583	9.951	13.525	83.23	99.59	93.75
95.8	5.584	9.951	13.535	83.18	99.59	93.77
96.2	5.587	9.951	13.546	83.12	99.59	93.78
97.1	5.589	9.951	13.558	83.07	99.60	93.79
99.7	5.592	9.951	13.571	83.01	99.60	93.81
104.5	5.595	9.952	13.585	82.94	99.60	93.83
109.8	5.599	9.953	13.598	82.88	99.61	93.85

111.5	5.602	9.952	13.610	82.82	99.62	93.86
116.6	5.606	9.953	13.622	82.76	99.63	93.88
121.9	5.609	9.952	13.635	82.71	99.62	93.89
124.9	5.613	9.952	13.642	82.67	99.63	93.91
129.9	5.616	9.953	13.654	82.61	99.64	93.93
134.7	5.620	9.951	13.665	82.57	99.64	93.94
137.8	5.623	9.951	13.676	82.52	99.65	93.95
140.2	5.627	9.950	13.686	82.48	99.65	93.97
139.7	5.630	9.951	13.697	82.44	99.65	93.99
145.4	5.636	9.949	13.715	82.37	99.66	94.00
148.1	5.638	9.949	13.721	82.34	99.67	94.01
150.8	5.641	9.949	13.728	82.32	99.68	94.02
155.1	5.645	9.948	13.740	82.27	99.67	94.03
158.6	5.651	9.948	13.751	82.22	99.69	94.05
161.9	5.656	9.946	13.763	82.18	99.70	94.06
164.7	5.659	9.945	13.773	82.14	99.70	94.07
168.0	5.663	9.944	13.782	82.11	99.71	94.07
171.5	5.667	9.944	13.790	82.07	99.72	94.08
175.2	5.671	9.943	13.801	82.03	99.73	94.09
178.8	5.676	9.942	13.810	82.00	99.72	94.10
182.0	5.680	9.941	13.821	81.97	99.73	94.11
185.3	5.684	9.939	13.831	81.94	99.75	94.12
188.5	5.688	9.937	13.841	81.90	99.75	94.12
191.9	5.693	9.936	13.851	81.87	99.76	94.13
195.7	5.697	9.936	13.861	81.84	99.77	94.14
198.3	5.702	9.932	13.872	81.81	99.77	94.15
202.1	5.706	9.931	13.882	81.79	99.79	94.15
205.7	5.711	9.929	13.893	81.75	99.80	94.15
209.1	5.715	9.928	13.901	81.73	99.80	94.16
212.2	5.719	9.927	13.910	81.70	99.82	94.16
215.5	5.724	9.925	13.919	81.68	99.83	94.16
219.4	5.728	9.922	13.930	81.66	99.84	94.16
222.8	5.733	9.920	13.943	81.63	99.85	94.16
225.5	5.738	9.917	13.953	81.62	99.86	94.16
229.3	5.742	9.916	13.963	81.58	99.86	94.16
232.4	5.747	9.914	13.973	81.56	99.87	94.17
235.8	5.752	9.909	13.988	81.56	99.90	94.15
239.2	5.757	9.907	14.000	81.53	99.91	94.15
243.1	5.762	9.904	14.010	81.51	99.91	94.15
246.5	5.766	9.903	14.021	81.48	99.92	94.16
249.6	5.770	9.900	14.030	81.47	99.92	94.15
253.0	5.776	9.892	14.050	81.49	99.97	94.10

Table S5. Values (α_X) of the principal axis of thermal expansion (X_1, X_2 and X_3) and their orientation in regards of the cell axis, a, b and c of C_4F_9-NDI Form α.

	Principal axis	α_X (MK^{-1})	a	b	c
Form α	X_1	-79	0.1583	0.9250	-0.3454
	X_2	184	0.9955	-0.0135	0.0939
	X_3	287	-0.0673	0.5127	0.8559
	Volume	398			

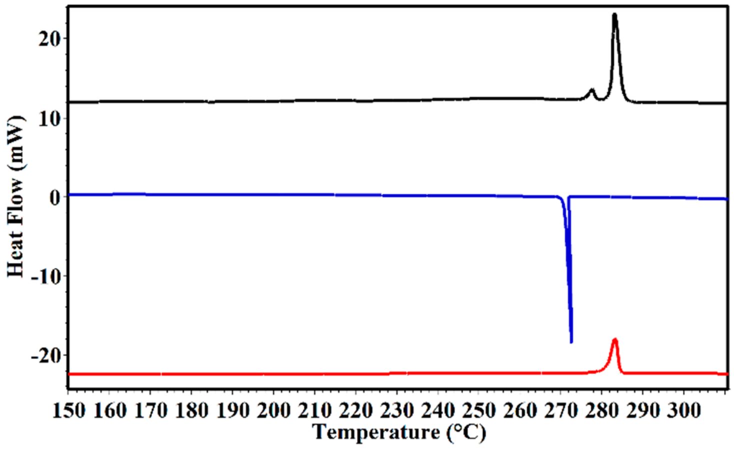

Figure S12. DSC of C_4F_9-NDI Form α which shows the non-reversible transition to C_4F_9-NDI Form β.

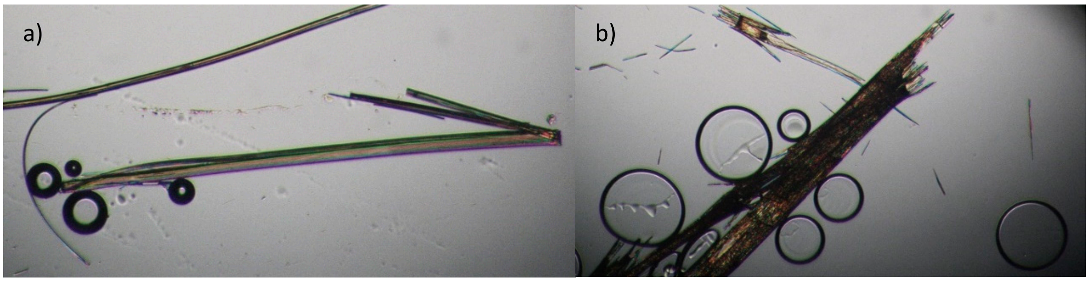

Figure S13. a) Desolvation of C_3F_7-NDI·ACN and b) C_4F_9-NDI·ACN.
